# Non‐Invasive Phenotyping of Sugar Beet and Maize Roots Using Field‐Scale Spectral Electrical Impedance Tomography

**DOI:** 10.1111/pce.70049

**Published:** 2025-07-06

**Authors:** Valentin Michels, Maximilian Weigand, Lena Lärm, Onno Muller, Andreas Kemna

**Affiliations:** ^1^ Institute of Geosciences, Geophysics Section University of Bonn Bonn Germany; ^2^ Institute of Bio‐ and Geosciences, Agrosphere (IBG‐3), Forschungszentrum Jülich Jülich Germany; ^3^ Institute of Bio‐ and Geosciences, Plant Sciences (IBG‐2), Forschungszentrum Jülich Jülich Germany

**Keywords:** agrogeophysics, induced polarization, maize, root phenotyping, spectral electrical impedance tomography, sugar beet

## Abstract

Root systems are essential for plant water and nutrient uptake, but are difficult to characterize in‐situ due to their inaccessibility. Spectral electrical impedance tomography (sEIT) is a non‐invasive geoelectrical method that has shown potential to quantify root traits at the laboratory scale. However, field applications remain scarce due to technical limitations and challenges in separating soil and root polarization signatures. This study explores the use of sEIT for in‐situ phenotyping of sugar beet and maize root systems. We conducted multi‐frequency sEIT measurements at varying crop growth stages to derive the subsurface complex resistivity distribution. Spectral analysis revealed high‐frequency polarization peaks for both species. Additionally, sugar beets exhibited an additional low‐frequency peak late‐season, which we attribute to the development of large storage parenchyma. For sugar beet, the high root‐to‐soil volume fraction allowed a direct correlation of electrical parameters to root biomass density. For maize, the superimposed soil polarization necessitated an alternative approach: We introduce an electrical root index (ERI) as a spectral dispersion measure indicative of root presence and show its correlation to root biomass density. Our findings demonstrate that sEIT is sensitive to macro‐ and microscopic root traits under field conditions, holding great potential for non‐invasive phenotyping of plant roots.

## Summary statement

In our study, we employed spectral electrical impedance tomography as a non‐invasive tool for field root phenotyping of maize and sugar beet plants. We show that the electrical signatures correlate to both macro‐ and microscopic root traits, highlighting the potential for root trait quantification.

**Abbreviations**
DAS: Days after sowing
ERI: Electrical root index
RBD: Root biomass density
sEIT: Spectral electrical impedance tomography

## 1 Introduction

Root systems are vital to the productivity and yield of agricultural crops due to their significance for water and nutrient uptake processes (Lynch 1995). Therefore, the characterization of root systems is of great importance for phenotyping, that is, the breeding of plants with optimized traits (e.g., Lynch 2007). For example, plants were grown with enlarged cortical cell size (Chimungu et al. 2014) to increase yield under drought conditions. Similarly, crops were adapted with deeper root systems to improve late season water availability (Wasson et al. 2014). Although root research has accelerated in recent years, there still exists a gap between above‐ground and below‐ground phenotyping methods, as the latter has developed much slower due to the inaccessibility of the rhizosphere (e.g., Watt et al. 2020). There is a particular lack of non‐invasive, in‐situ phenotyping methods, which are needed as an alternative characterization tool to conventional invasive and labour‐intensive methods, such as root coring (e.g., Burridge et al. 2020), trenching (e.g., Teramoto and Uga 2020), or shovelomics (e.g., Trachsel et al. 2011). Non‐invasive root phenotyping on the lab‐scale has made significant advancements through the employment of modern imaging techniques, like X‐ray computed tomography (e.g., Teramoto et al. 2020; Herrero‐Huerta et al. 2021), neutron radiography (e.g., Oswald et al. 2008), magnetic resonance imaging (e.g., Pflugfelder et al. 2017), and advanced high‐throughput phenotyping platforms (e.g., Gioia et al. 2017; Martins et al. 2020; Falk et al. 2020; Liu et al. 2021). However, although attempts are being made to transfer these techniques to the field (e.g., Bagnall et al. 2022), and progress has been made in developing plot‐scale setups such as minirhizotrons (e.g., Lärm et al. 2023), their application is, to date, often limited to smaller scales or controlled laboratory conditions.

Geoelectrical methods are able to non‐invasively map electrical subsurface properties using injected electrical currents, and since they are not limited to lab‐scale investigation volumes, they hold great potential for field root research (e.g., Cimpoiaşu et al. 2020). Electrical resistivity tomography (ERT), a well‐established method that uses an array of spatially distributed electrodes to reconstruct 2D or 3D resistivity distributions of the subsurface (e.g., Binley and Slater 2020), has been used in a multitude of studies to assess root water uptake dynamics of various crops at the plot scale (e.g., Srayeddin and Doussan 2009; Garré et al. 2011, 2012; Whalley et al. 2017; Blanchy et al. 2020; Vanella et al. 2022; Chou et al. 2024). Beyond root water uptake, ERT has been used to examine the effects of agricultural practices and soil compaction on soil water distribution (Carrera et al. 2022, 2024), and more recently, Blanchy et al. (2025) transformed resistivity variations in an extensive field monitoring experiment to actionable phenotyping indicators for plant scientists and breeders. While these studies primarily used resistivity changes to indirectly infer root system activity, others utilized ERT to directly quantify the biomass of tree roots (Amato et al. 2008, Rossi et al. 2011) and fine root systems (Amato et al. 2009). A specialized form of ERT, called mise‐à‐la‐masse, injects current directly into the stem of the plant, allowing the localization of current pathways in the root system. Through inversion of the current source density, researchers have been able to provide information about the dimensions and architecture of vine (Mary et al. 2020; Mary et al. 2023) and maize plants (Peruzzo et al. 2020) at the individual plant scale. However, problems with current leakage near the plant stem were reported that hindered delineation of the root system size (Urban et al. 2011; Peruzzo et al. 2020). Although resistivity surveys have proven its value for root research, interpretation can be highly ambiguous due to sensitivity of resistivity to numerous environmental factors (e.g., Binley and Slater 2020). Fine root growth, in particular, may be difficult to distinguish from changes in soil temperature, moisture, or fluid conductivity without auxiliary data (e.g., Ehosioke et al. 2020).

Another group of geoelectrical methods that is increasingly being used for root research are induced polarization (IP) methods, as they can help reduce said ambiguities through the additional measurement of polarization effects in the subsurface (e.g., Kessouri et al. 2019). For root research, IP methods are commonly employed utilizing an alternating injection current at varying frequencies in the mHz to kHz range (also referred to as frequency‐domain IP) to infer additional information about the polarizability of a material (e.g., Binley and Slater 2020). At frequencies below 10 kHz, polarization effects in porous media are mainly caused by so‐called electrical double layers (EDLs), ion concentration gradients that form at charged surfaces such as soil particles (e.g., Lyklema et al. 1983; Bücker et al. 2019) or root cell membranes (e.g., Prodan et al. 2008; Kessouri et al. 2019). From the characteristic relaxation time that ions require to move back into an equilibrium state after disturbance by an electric field, manifested in the spectral polarization signature, information can be gained about characteristic length scales in the medium, for example pore throat diameter (e.g., Binley et al. 2005; Titov et al. 2010) or root cell size (e.g., Weigand and Kemna 2019). Polarization effects in roots were first observed by Chloupek (1972) using two‐electrode capacitance measurements, and many studies have since reported a dependence of the capacitance to a variety of root traits under laboratory and field conditions, for example root biomass (e.g., Kendall et al. 1982; Dalton 1995; Ozier‐Lafontaine and Bajazet 2005; Cseresnyés et al. 2018; Středa et al. 2020), root surface area (e.g., Cseresnyés et al. 2021), root length (e.g., Ellis et al. 2013), and even root activity (e.g., Cseresnyés et al. 2024a, 2024b). Similarly, the polarization signature of four‐point electrical impedance measurements of root systems could be correlated to root biomass and surface area (e.g., Tsukanov and Schwartz 2020), root length (e.g., Peruzzo et al. 2021), and the cross‐sectional area of root segments (Ehosioke et al. 2023).

The drawback of a single, integral capacitance or four‐point impedance measurement is the inability to image the 2D or 3D distribution of electrical properties of a whole root system, which requires a multitude of spatially differing four‐point measurements within the area of interest (e.g., Binley and Slater 2020). Inference of the spatially resolved conduction and polarization properties is then achieved using tomographic inversion algorithms (e.g., LaBrecque et al. 1996; Kemna 2000; Wang et al. 2023a). So far, studies dealing with the tomographic imaging of root polarization signatures are still scarce, and have mostly been performed under hydroponic laboratory conditions. Weigand and Kemna (2017) were the first to use spectral electrical impedance tomography (sEIT), the IP‐equivalent to ERT, to spatially reconstruct the polarization signature of barley roots, and additionally showed the method's sensitivity to the physiological state of the plant. Weigand and Kemna (2019) linked the diurnal variation in polarization strength of barley root to artificial day‐night cycles. Another study used high‐frequency ( ≥ 10 kHz) sEIT to reconstruct the shape of potato, carrot, and peony roots (Wang et al. 2023b). More recently, Michels et al. (2024) quantified root biomass and surface area of various root systems with the tomographic polarization signature obtained from sEIT measurements. Scaling up sEIT to the field application poses various challenges to the measurement design, including the choice of electrodes, influence of environmental parameters, and the consideration of capacitive and inductive effects of the setup (e.g., Kelter et al. 2018). In combination with the increased measurement time and more expensive and complex instrumentation (compared to ERT), these challenges have long hindered application of the method to field root research. However, recent progress in instrumentation (e.g., Zimmermann et al. 2008; Flores Orozco et al. 2021) and processing pipelines to correct for electromagnetic coupling of measurement cables (e.g., Zhao et al. 2015) and high‐frequency leakage currents (Zimmermann et al. 2019), as well as the investigation of the effect of heterogeneous contact impedances of electrodes on phase readings (Zimmermann and Huisman 2024), have enabled robust field‐scale sEIT surveys with measurement frequencies of up to 1 kHz. Weigand et al. (2022), for example, described a long‐term sEIT monitoring setup and associated analysis procedures, resulting in robust multi‐month imaging results for frequencies up to 1 kHz.

In this study, we explore the feasibility of using sEIT to image root systems at the field scale. For this, we designed an experiment with sEIT measurements over three timesteps of the growing season, targeting the contrasting root architectures of sugar beet (larger taproot) and maize (finer fibrous roots). Here, we focus on the following research questions:

1. Can we measure root polarization signatures up to 1 kHz at the field scale, and if so, do large targets like sugar beet exhibit different polarization behavior compared to fine root systems like maize?
2. Can we differentiate between the electrical response of soil, maize roots, and sugar beet?
3. Can we extract root traits, for example, root biomass density, from the electrical measurements, and can we use the same procedure for both sugar beet and maize root traits?

With this study, we aim to improve the current understanding of in‐situ polarization signatures of root systems using state‐of‐the‐art measurement instrumentation and processing procedures for sEIT measurements.

## 2 Materials & Methods

### 2.1 Spectral Electrical Impedance Tomography

sEIT surveys are usually conducted with a set of four‐electrode measurement configurations, where two electrodes (A, B) are utilized for current injection, and the other two (M, N) for measuring the resulting potential difference (voltage) associated with the generated electric field (e.g., (Binley and Slater 2020). From the (complex‐valued) voltage ${\hat{U}}_{\text{MN}}(\omega )$ and injected current ${\hat{I}}_{\mathrm{AB}}(\omega )$, the transfer impedance $\hat{Z}(\omega )$ can be computed for varying injection frequencies ω, typically ranging from a few mHz to kHz:

(1) $$\hat{Z}(\omega )=\frac{{\hat{U}}_{\mathrm{MN}}(\omega )}{{\hat{I}}_{\mathrm{AB}}(\omega )}.$$

Here, the geometric alignment of the four electrodes, represented by the so‐called geometric factor, influences the sensitivity to areas in the subsurface and can be used to estimate average, non‐localized complex conductivity values (also referred to as apparent conductivity; see, for example, Binley and Slater 2020) from the impedance data. The spatial reconstruction of the complex conductivity $\hat{\sigma }(\omega )$ of the subsurface can be achieved using numerical inversion schemes, which involves discretization of the subsurface into a grid of parameter cells. For an overview and comparison of different inversion approaches, the reader is referred to Wang et al. (2023a). The complex conductivity (or its inverse, the complex resistivity $\hat{\rho }(\omega )$) is usually expressed in real and imaginary components,

(2) $$\hat{\sigma }(\omega )=\frac{1}{\hat{\rho }(\omega )}=\sigma^{\prime }(\omega )+i\sigma^{\prime \prime }(\omega ),$$

or in polar notation:

(3) $$\hat{\sigma }(\omega )=|\hat{\sigma }(\omega )|e^{i\varphi_{\sigma }}.$$

Here, the real part $\sigma^{\prime }(\omega )$ or magnitude $\left|\hat{\sigma }(\omega )\right|$ describes the conduction properties, while the imaginary part $\sigma^{\prime \prime }(\omega )$ or phase shift $\varphi_{\sigma }$ describes the polarization properties of the medium. In the inversion, uncertainties of the measurement data are commonly described using error models. For the impedance magnitude, we assume a linear error model (LaBrecque et al. 1996):

(4) $$\Delta |\hat{Z}(\omega )|=a|\hat{Z}(\omega )|+b,$$

where $\left|\Delta \hat{Z}(\omega )\right|$ is the impedance magnitude error, a is the relative and b the absolute impedance magnitude noise level. Due to the utilized irregular measurement scheme (Section 2.4.1), we were not able to record reciprocal measurements that would allow implementation of more sophisticated phase error models (e.g., Orozco et al. 2012). Instead, similar to the impedance magnitude, a linear error model was assumed for the phase error $\Delta \varphi_{Z}$:

(5) $$\Delta \varphi_{Z}=c\varphi_{Z}+d,$$

with $\varphi_{Z}$ being the impedance phase shift, c the relative, and d the absolute phase error estimate.

### 2.2 Spectral Analysis

Often, it is beneficial to evaluate the polarization behaviour of a material over a wider range of frequencies to capture polarization processes at multiple scales. To do so, empirical models are employed that can describe the obtained signature with integral electrical parameters. In this study, we used the Debye decomposition scheme (Nordsiek and Weller 2008; Weigand and Kemna 2016), a model that describes the measured complex resistivity spectra using a number of Debye relaxation terms:

(6) $$\hat{\rho }(\omega )=\rho_{0}\left(1-\sum_{k=1}^{N}m_{k}\left[1-\frac{1}{1+i\omega \tau_{k}}\right]\right),$$

with $\rho_{0}$ being the (real‐valued) direct‐current resistivity, $m_{k}$ the k th chargeability, and $\tau_{k}$ the k th relaxation time for the k th Debye relaxation term. From this model, the total chargeability $m_{\text{tot}}$, a measure of the polarization strength, can be computed:

(7) $$m_{\mathrm{tot}}=\sum_{k=1}^{N}m_{k}.$$

Additionally, the mean relaxation time $\tau_{\text{mean}}$ is retrieved, which gives information about the average polarization time scales in the medium:

(8) $$\tau_{\mathrm{mean}}=\exp(\sum_{k=1}^{N}m_{k}\log(\tau_{k})⁄\sum_{k=1}^{N}m_{k}).$$

Since the polarization time scales usually correlate with the characteristic length scales of the underlying polarization process, information such as grain size (e.g., Schwarz 1962; Lyklema et al. 1983), pore throat diameter (e.g., Binley et al. 2005; Titov et al. 2010; Revil et al. 2012), or root cell diameter (Weigand and Kemna 2017, 2019), can be inferred. After Schwarz (1962), the relaxation time for electrochemical (ionic) polarization processes around colloidal particles is described by

(9) $$\tau =\frac{r^{2}}{2D},$$

with r being the particle radius, and D the diffusion coefficient.

### 2.3 Extraction of Root Polarization Signatures

In this study, we used sEIT to investigate sugar beet and maize roots as two structurally and physiologically differing biological targets. Sugar beet roots are larger, with dimensions of multiple decimeters, making them spatially resolvable as an electrical anomaly on their own (provided the electrode spacing is sufficiently small). In contrast, the fine root system of maize cannot be resolved individually, and the measured electrical response must be interpreted as a compound signature of roots and soil. We therefore propose to employ different analysis procedures for both plant types. For sugar beet, we derive the chargeabilities and relaxation times for all cells that lie in the vicinity of the plant positions, under the assumption that most of the captured signatures have their origin in the beet polarization. Analogous to Michels et al. (2024), we aim to compare the average chargeability ($\overset{-}{m}$) in the root zone, weighted by the volume of the corresponding cells in the inversion grid (i.e., parameter cells forming the tomographic image), with the beet root biomass density ($RBD_{\mathrm{sb}}$), and seek to relate the average relaxation time ($\overset{-}{\tau }$) of dominant polarization processes to characteristic length scales (λ) within the root:

(10) $$\overset{-}{m}=\frac{1}{V_{\mathrm{rz}}}\sum_{j=1}^{n}V_{j}m_{\text{tot},j}∼RBD_{\mathrm{sb}}=\frac{M_{\mathrm{root},\mathrm{sb}}}{V_{\mathrm{rz}}},$$

(11) $$\overset{-}{\tau }=\frac{1}{V_{\mathrm{rz}}}\sum_{j=1}^{n}V_{j}\tau_{\text{mean},j}∼\lambda^{2}.$$

Here, $m_{\text{tot},j}$ represents the total chargeability, $\tau_{\text{mean},j}$ the mean relaxation time, $M_{\text{root},\text{sb}}$ the sugar beet root biomass, $V_{j}$ the cell volume of the j th grid cell, and $V_{\text{rz}}=\sum V_{j}$ the total volume of the region defined as “root zone” (in this study, the soil‐root volume excavated for validation, see Section 2.4.2 below).

For maize, this procedure is not feasible: Unlike in studies that estimate the root polarization under hydroponic conditions (e.g., Weigand and Kemna 2017, 2019; Tsukanov and Schwartz 2020; Michels et al. 2024), where correlations between polarization strength and root traits may be directly established, the superimposed polarization of the background medium inhibits the direct analysis of the spectral response. As this background polarization is also not static, but changes dynamically with variations in for example temperature (e.g., Bairlein et al. 2016) and soil water content (e.g., Breede et al. 2012; Kelter et al. 2018), an analysis procedure is needed that minimizes these effects, while simultaneously gaining information about the roots in the investigation volume.

**Figure A1:**
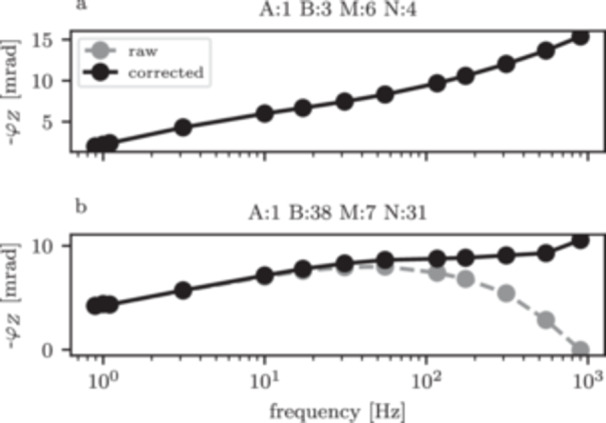
Exemplary impedance phase spectra for data that are practically uninfluenced by inductive effects (a) and data that show strong high‐frequency inductive effects (b). The used electrode numbers A, B, M, N for each four‐point measurement are noted in the subfigure titles. Raw spectra are shown as dashed grey lines, spectra after correction for inductive effects in black. [Color figure can be viewed at wileyonlinelibrary.com]

Previous studies have shown that the polarization of fine root systems is frequency dependent and significantly stronger in higher (≥ 1 kHz) frequency ranges. For example, Ehosioke et al. (2023) found the strongest polarization for maize root segments in their experiments to be between 10 and 40 kHz, and similar ranges were observed for wheat and pecan plants by Peruzzo et al. (2021). More recently, Gu et al. (2024) found that the correlation of capacitance with root biomass increases with the employed measurement frequency. Consequently, we expect that the maize roots in our field experiment will have little impact on the polarization signature at lower frequencies, while significantly contributing to the high‐frequency polarization. In contrast to this, soil water content and temperature have been shown to affect both low‐ and high‐frequency ranges (e.g., Jougnot et al. 2010; Breede et al. 2012; Bairlein et al. 2016; Revil et al. 2023). We therefore propose using the slope of the spectral polarization signature between high‐frequency (mixed root‐soil) and low‐frequency (predominantly soil) response, that is, a measure of the spectral dispersion, as an indicator for root presence (for an example polarization spectrum, see Figure A1). This is based on the hypothesis that changes in this electrical quantity primarily reflect variations associated with root growth. We refer to this indicator as electrical root index (ERI), which is computed from the differential quotient of high‐frequency ($f_{\text{HF}}$) and low‐frequency ($f_{\text{LF}}$) logarithmic imaginary parts of the complex conductivity spectrum ($\sigma_{\mathrm{HF}}^{\prime \prime }$ and $\sigma_{\mathrm{LF}}^{\prime \prime }$, respectively):

(12) $$ERI=\frac{\log(\sigma_{\mathrm{HF}}^{\prime \prime })-\log(\sigma_{\mathrm{LF}}^{\prime \prime })}{\log(f_{\text{HF}})-\log(f_{\text{LF}})}=\frac{\log(\frac{\sigma_{\mathrm{HF}}^{\prime \prime }}{\sigma_{\mathrm{LF}}^{\prime \prime }})}{\log(\frac{f_{\text{HF}}}{f_{\text{LF}}})}.$$

For small phase values, that is, $\sigma^{\prime \prime }=|\hat{\sigma }|sin(\varphi )\approx |\hat{\sigma }|\varphi$, and $\left|{\hat{\sigma }}_{\mathrm{HF}}|\approx |{\hat{\sigma }}_{\mathrm{LF}}\right|$, one can alternatively express the ERI as

(13) $$ERI\approx \frac{\log(\frac{\varphi_{\mathrm{HF}}}{\varphi_{\mathrm{LF}}})}{\log(\frac{f_{\text{HF}}}{f_{\text{LF}}})}.$$

We hypothesize that this procedure dampens the effect of soil water, nutrient, and temperature variations on the polarization signature of the soil, yielding a quantity primarily sensitive to changes in root content. There are several assumptions that are going into this approach: First, we assume that the electrical soil signature is comparable between the three sEIT measurement profiles, and does not change significantly with depth. Secondly, we assume that factors like temperature and soil water content affect all frequencies similarly, and variations between frequencies are small in comparison to the influence of the root polarization.

Based on these ideas, we expect a correlation between the average, cell‐volume‐weighted electrical root index $\bar{\mathrm{ERI}}$ of all cells near the maize plant positions, and the maize root biomass density ($RBD_{m}$) in this volume:

(14) $$\bar{ERI}=\frac{1}{V_{\mathrm{rz}}}\sum_{j=1}^{n}V_{j}ERI_{j}∼RBD_{m}=\frac{M_{\mathrm{root},m}}{V_{\mathrm{rz}}}.$$

Here, $ERI_{j}$ denotes the electrical root index of the j th cell in $V_{\text{rz}}$. As low‐ and high‐frequency limits for the ERI, we chose 3.125 Hz and 1 kHz, respectively, related to the magnitude of retrieved phase values in the experiment (close to zero at some locations in the profile when using frequencies ≤ 3.125 Hz), and technical constraints of the utilized measurement device.

### 2.4 Experimental Setup

**Figure 1:**
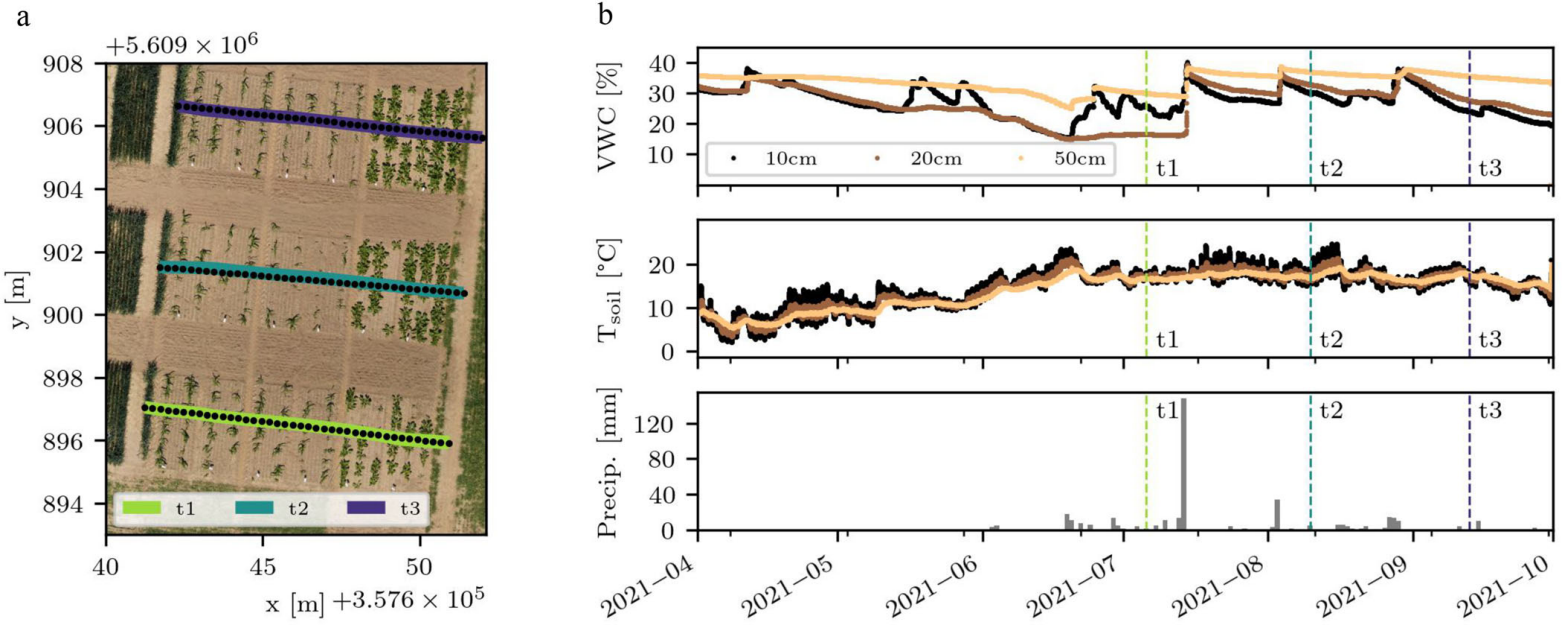
(a) Overview of the measurement site with marked profile locations. Electrode positions are displayed as black points, geographical coordinates are shown in UTM32N coordinates. The background image was taken on the 14th of June 2021 (Source: Erekle Chakhvashvili, Campus Klein Altendorf Central Experiment RGB orthomosaics 2021, https://doi.org/10.48574/4jq2‐9y53). (b) Volumetric soil water content and soil temperature for different depths, and daily precipitation throughout the season. sEIT measurement timesteps t1, t2, t3 are marked as dashed vertical lines.

The experiment was conducted on sugar beet (*Beta vulgaris*, variety “BTS 440”) and maize (*Zea mays*, variety “Sweet Nugget”) plots in the summer of 2021 at the field site Campus Klein‐Altendorf near Meckenheim, Germany. Sugar beet and maize were sown on 1st and 26th of April 2021, with crop row spacings of 40 and 75 cm, respectively. To characterize root development over time, we conducted sEIT measurements in three spatially varying profiles throughout the growing season (6th of July, 10th of August and 13th of September, labeled as t1, t2 and t3, respectively). Using 40 stainless steel electrodes with a length of 17 cm and diameter of 1 cm, profiles were set up with an electrode spacing of 25 cm (i.e., a total profile length of 9.75 m). Electrodes were drilled approximately 14–15 cm into the soil – an overview of the placement for all three profiles can be seen in Figure 1a. Similarly to Weigand et al. (2022), all but the lower 5 cm of each electrode was isolated with rubber tubing to approximate a point electrode that is assumed in the inversion of the data. We used shielded S/FTP Cat6 Ethernet cables and elevated them approximately 10 cm from the ground with Styrodur blocks and rain gutters to prevent leakage of injected current into the soil through cable walls (Weigand et al. 2022).

Soil sampling at the site was performed with a Pürkhauer auger to a depth of 90 cm, and after being oven‐dried at 104°C for 2 days, the particle distribution was analyzed by sieving and sedimentation according to DIN ISO 11277. The soil texture can be classified into three distinct horizons: The upper layer (0–30 cm) contains 15.2%–18.5% clay, 72.5%–75.5% silt, and 6.0%–7.5% sand. The second layer (30–60 cm) is similar in clay content, but has slightly higher silt‐content (75.5%–78.1%) and a correspondingly reduced sand fraction (4.5%–6.0%). The lower layer (60–90 cm) is generally higher in clay content, averaging values of 25.1%–28.4% clay, 66.5%–69.5% silt, and 4.5%–6.0% sand. Soil temperature and water content (Figure 1b) were monitored using sensors of the SoilNet network (Bogena et al. 2010) in a different experiment right next to the measurement profiles. Precipitation data (Figure 1b) were recorded at a weather station on‐site and downloaded from the website of the agricultural meteorology Rhineland Palatinate (https://www.wetter.rlp.de/). The growing season in 2021 was defined by an initial dry period until mid June, followed by a longer stretch of wet days with exceptionally heavy precipitation events, most notably in July (Ludwig et al. 2023). Soil water content increased from timestep t1 to t2 in all recorded depths, and slightly decreased towards t3. Soil temperatures at the measurement days ranged between 16 and 17 C in the lower soil layers and were slightly more variable at 10 cm depth, related to weather conditions on the respective measurement days.

#### 2.4.1 sEIT Data Acquisition and Processing

The sEIT surveys were performed at 23 logarithmically evenly spaced frequencies from 0.1 Hz to 10 kHz using the EIT40 impedance tomograph developed by Zimmermann et al. (2008). For each current injection dipole, the device records the electrical potential at all remaining electrodes. From the obtained three‐point measurements, arbitrary four‐point configurations can be computed by superposition. One survey consisted of 70 current injections, with most dipoles between an electrode skip of 20 and 22, and took approximately 3 h and 15 min to complete. To maximize information content, we generated a measurement scheme with all possible potential dipoles for the used current injections, and limited the selection only by a maximum geometric factor of 5 m. This approach was chosen because configurations with small geometric factors typically have a high signal‐to‐noise ratio, which is crucial for ensuring sufficient data quality in sEIT measurements (Weigand et al. 2022). Additionally, we removed configurations in which the potential electrodes were used as current electrodes in the previous injection to avoid electrode polarization effects (e.g., Dahlin 2000). The final scheme consisted of 9104 configurations.

Following the approach in Zhao et al. (2015) and Weigand et al. (2022), the raw datasets were corrected for high‐frequency inductive effects between measurement cables. However, since no calibration measurement was performed during the surveys, we solely relied on the numerically‐determined mutual cable inductances for correction. This may have introduced uncertainties due to the unknown exact cable locations that are assumed in the numerical simulation (Weigand et al. 2022). Still, we expect these deviations to be smaller than the influence of inductive effects if no correction would be performed at all. Two exemplary raw and corrected impedance phase spectra from timestep t1 are visualized in the Appendix in Figure A1. While the spectrum shown in Figure A1a does not exhibit strong inductive effects, and therefore does not change significantly after the correction procedure, the spectrum in Figure A1b shows considerable high‐frequency effects in the raw data.

Overall, data quality was good, with electrode contact resistances ranging between 600 Ω and 2 kΩ. Leakage currents (i.e., current i.e. exiting the measurement system through other points than the electrodes, e.g. cable walls) ranged between 0.01% and 1% of the injected current, indicating low current loss through the experimental setup. For each frequency, we removed positive phase values above 5 mrad, and subsequently filtered the data by removing all configurations where deviations from the mean impedance phase exceeded three times the respective standard deviation. Although measurements were performed with frequencies up to 10 kHz, we chose to only invert datasets up to a frequency of 1 kHz, as phase measurement accuracy of the EIT40 decreases at higher frequencies due to electromagnetic coupling effects (Zimmermann et al. 2008).

For all frequencies from 0.1 Hz to 1 kHz, the filtered data were inverted using the complex resistivity inversion code CRTomo (Kemna 2000). We assumed an absolute impedance magnitude error of 0.01 Ω, and relative impedance magnitude error of 2%. With an absolute phase error set to half of the standard deviation of the phase values for each inverted frequency, and a relative phase error of 2%, we achieved convergence of all inversions to an error‐weighted RMS (root mean square) data misfit of 1, indicating that the measurement data were predicted by the final model realization within the assumed data error estimates (e.g., Kemna 2000). To analyze the spectral polarization behaviour, we performed a Debye decomposition with the obtained complex resistivity spectra for each cell to estimate the total polarization strength and polarization lengthscales.

#### 2.4.2 Validation of Imaging Results With Plant Traits

After each sEIT measurement, we collected root and shoot validation data for the electrical measurements using “shovelomics” (Trachsel et al. 2011), a high‐throughput phenotyping method in which the upper root system of the plant is excavated with a shovel. We dug out a soil and root volume of 20 × 20 × 25 cm^3^ around each maize plant stem, and the root systems were subsequently carefully washed with regular tap water on‐site. We estimated shoot height, fresh shoot biomass, and fresh root biomass for each maize plant along the profiles. The sugar beet plants were excavated in a similar manner and cleaned to remove any residual soil. Afterwards, the beet maximum diameter, length, as well as the fresh beet biomass were determined. Additionally, the phenological stages of the maize plants were categorized using the BBCH index (Meier 2003).

To analyze the polarization behavior of both plant types, we extracted the complex resistivity spectra of all grid cells in a 20 cm wide and 25 cm deep rectangular area around each plant position, corresponding to the excavated soil‐root volume described above. For each plant type, the complex resistivity of these cells was averaged to obtain a representative polarization signature. Additionally, we extracted and averaged spectra that we attributed mostly to the polarization of the soil from all cells at a depth of 50–100 cm below the maize plants. This is based on the assumption that in well watered conditions, which was the case in the 2021 season, the bulk of maize roots is commonly found in the upper 50 cm of soil (Sharp and Davies 1985), leaving deeper soil layers with only a small root fraction. Quantitative comparison of electrical polarization signatures with root traits was performed by using the equations in Section 2.3 on all cells within the excavated area, which we define as “root zone”. In particular, we compared resistivity magnitude and chargeability of both plant types to the root biomass density, and additionally compared the computed ERI in the maize plant area to the root biomass density.

## 3 Results

### 3.1 Complex Resistivity Imaging

**Figure A2:**
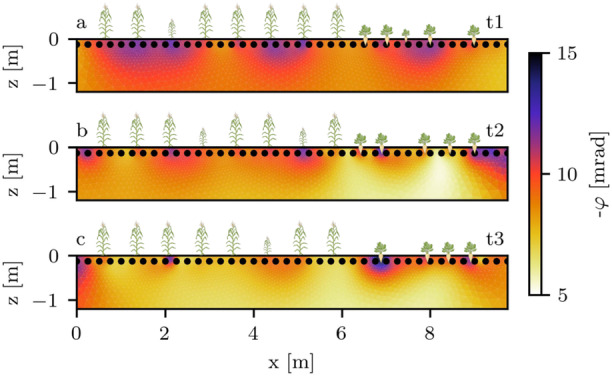
Resistivity phase imaging results at a measurement frequency of 175 Hz. Plant locations are indicated by maize (left) and sugar beet icons (right). Smaller icons indicate an off‐centered plant location (in direction perpendicular to the image plane). [Color figure can be viewed at wileyonlinelibrary.com]

In the following, we present the complex resistivity inversion results of all three profiles, given as magnitude and phase images, at measurement frequencies of 1 Hz and 1 kHz. Phase shift images of an additional intermediate measurement frequency (175 Hz) are given in the Appendix in Figure A2. Because the resistivity magnitude did not exhibit a strong spectral dependence in the investigated frequency range, it is only shown at a frequency of 1 Hz. Note that we excluded the outer areas of the inversion grid where measurement sensitivity is expected to be low, and the actual grid extended an additional 4 m to the left and right, and 2.8 m below the pictured section.

**Figure 2:**
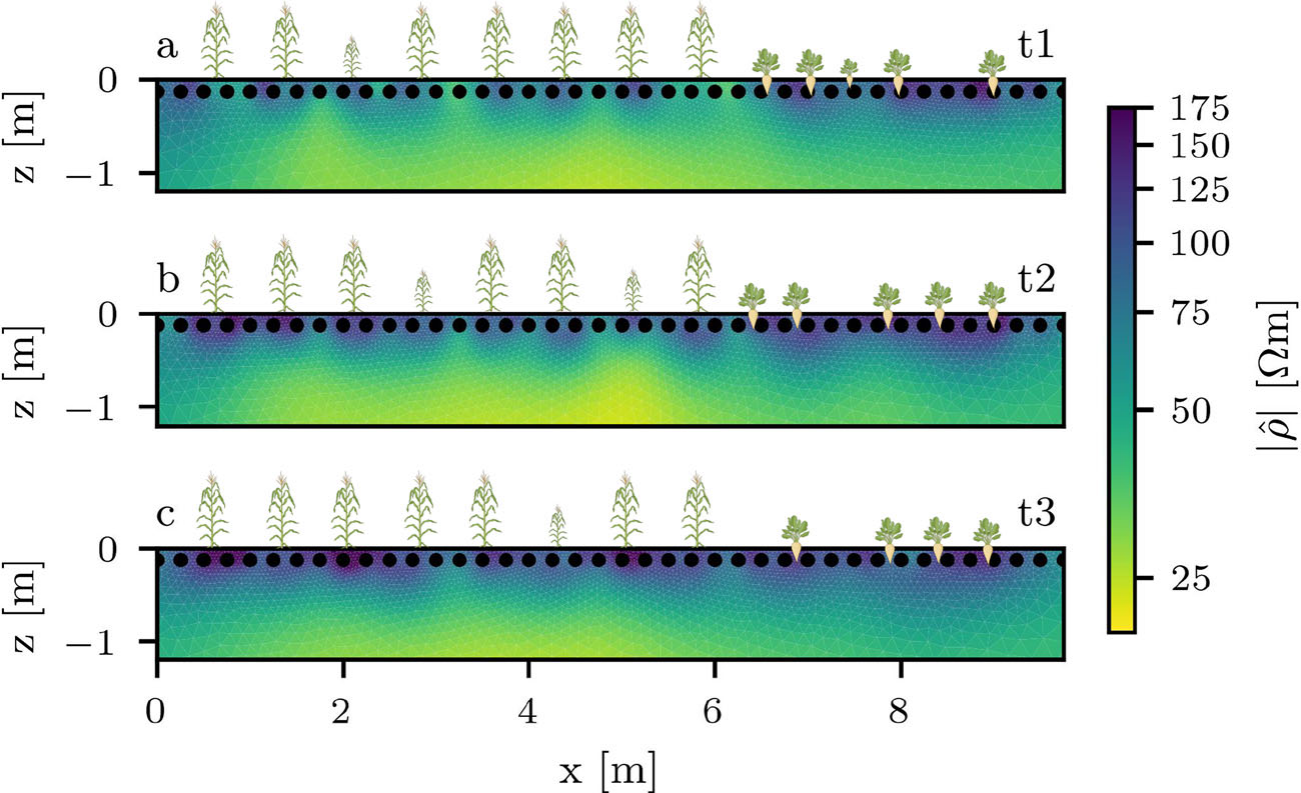
Complex resistivity magnitude images at 1 Hz for profiles t1, t2 and t3. Plant locations are indicated by maize (left) and sugar beet icons (right). Smaller icons indicate an off‐centered plant location (in direction perpendicular to the image plane). [Color figure can be viewed at wileyonlinelibrary.com]

The resistivity magnitude distribution for all timesteps varied between 25 and 150 Ωm (Figure 2). We observed high‐resistivity features near the plant locations – in contrast, lowest resistivity levels were found in deeper soil layers. These levels were lower for areas populated with maize compared to those with sugar beet. Comparing the different timesteps, a slight overall increase in resistivity magnitude could be observed in the maize plant area, while resistivity in the sugar beet area remained relatively constant across all timesteps. Additionally, resistivity values in the deeper soil layers below maize were lowest for timestep t2.

**Figure 3:**
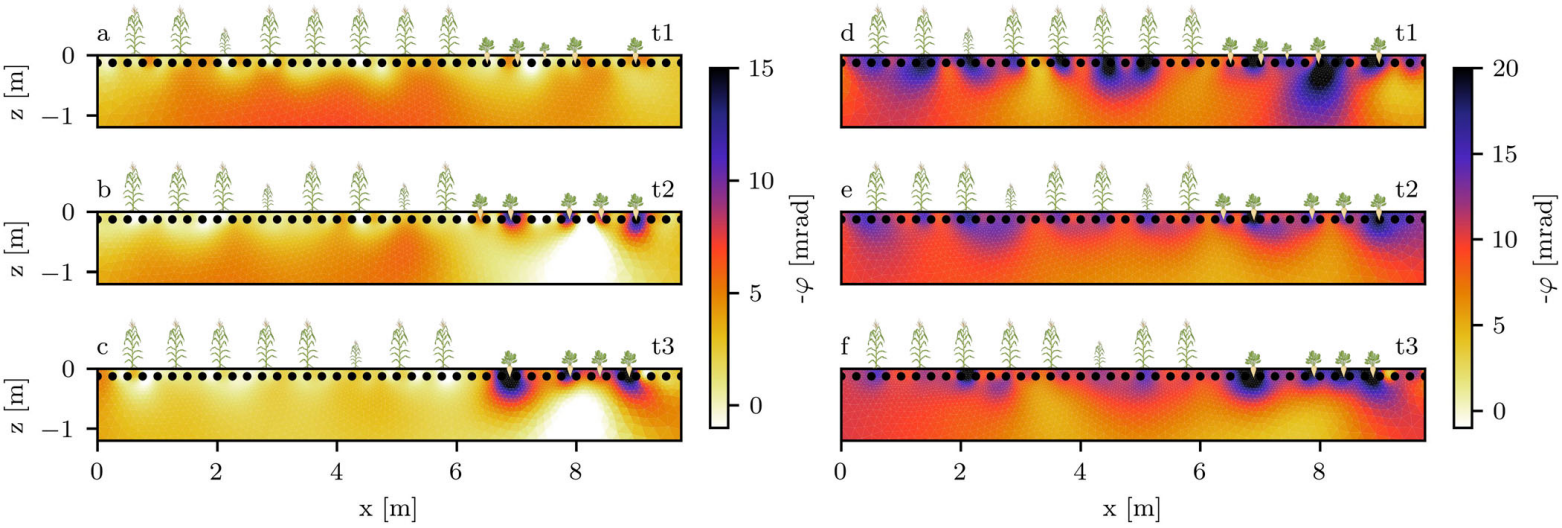
Complex resistivity phase images at 1 Hz (a‐c) and 1 kHz (d‐f) for profiles t1, t2, and t3. Plant locations are indicated by maize and sugar beet icons. Smaller icons indicate an off‐centered plant location (in direction perpendicular to the image plane). [Color figure can be viewed at wileyonlinelibrary.com]

The background phase values at lower frequencies (1 Hz, left column of Figure 3) averaged −7 mrad in timestep t1, and increased to maximum values of −1 mrad in timestep t3. Near the surface, we observed small anomalies where phase shifts were slightly higher in comparison to the background, or even positive. Near the maize plants, no significant polarization anomalies were found. However, while the sugar beet plants did not show significant polarization signatures at timestep t1, phase values decreased to below −15 mrad at the plant locations at later timesteps. Additionally, slightly positive phase anomalies were found below the area populated with sugar beet plants at timesteps t2 and t3.

At the highest inverted frequency (1 kHz, see right column of Figure 3), background phase values averaged approximately −10 mrad, and remained at a similar level for all timesteps. However, in comparison to the results at 1 Hz, decreased phase values, ranging from −15 to −25 mrad, were found at both the maize and sugar beet plant locations. Negative phase anomalies for the maize plants were strongest at the first and second timestep. At the sugar beet locations, phase values showed a general increase in magnitude towards the later timesteps.

#### 3.1.1 Spectral Electrical Soil and Root Signatures

**Figure 4:**
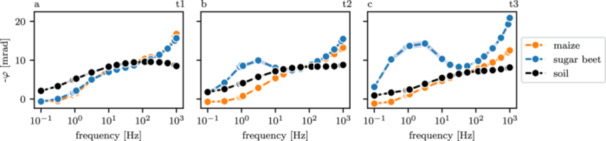
Average extracted resistivity phase spectra of the maize plant positions (orange), sugar beet positions (blue), and the soil (black) for timesteps t1 (a), t2 (b), and t3 (c). [Color figure can be viewed at wileyonlinelibrary.com]

The average complex resistivity spectra (see Section 2.4.2) revealed that for all three timesteps, the soil signature was characterized by a decrease in phase shift with increasing frequency, down to approximately −10 mrad (Figure 4). Overall, the shape of the spectra did not change significantly over time. Phase shifts near the maize plants also decreased with frequency, but started at lower phase values in the low‐frequency range and exhibited a steeper decrease toward higher frequencies (≥ 100 Hz), resulting in minimum phase values of approximately −15 mrad. In comparison to maize, sugar beet showed a distinctively different polarization behavior over the season: While the spectra were quite similar at the first timestep, a low‐frequency peak developed at timesteps t2 and t3. Additionally, the high‐frequency polarization was significantly stronger compared to the signature at the maize plant locations, with phase values ranging down to −25 mrad. The low‐frequency peak was not yet formed at the first timestep, and shifted its peak frequency towards lower frequencies over time (around 4–5 Hz at t2, and 1–2 Hz at t3). Additionally, the magnitude of the peak increased from −10 mrad at t2 to −15 mrad at t3.

**Figure 5:**
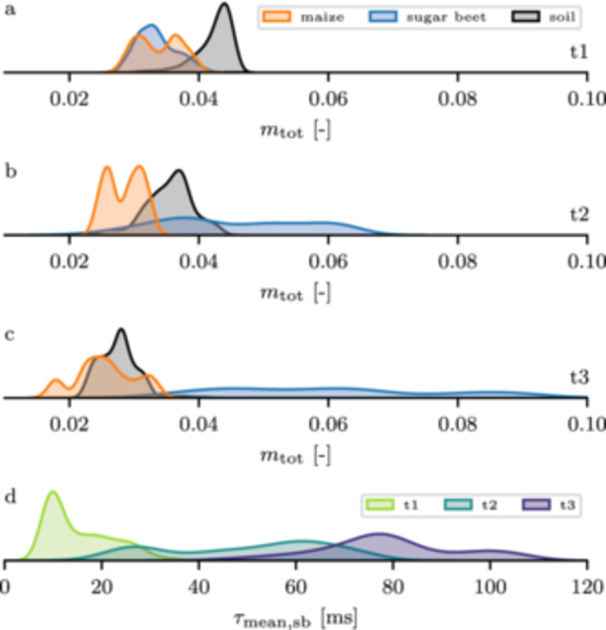
The upper three plots (a‐c) show the total chargeability distribution for maize (orange), sugar beet (blue), and root‐free soil (black) for all timesteps, retrieved from the full‐frequency Debye decomposition. The lower plot (d) shows the distribution of the mean relaxation time of the sugar beet plants for all timesteps, retrieved from the low‐frequency Debye‐decomposition. [Color figure can be viewed at wileyonlinelibrary.com]

Debye decomposition results of the individual grid cells (see Section 2.4.1) revealed that the overall total chargeability near the maize plants slightly decrased over the season, with values ranging from 0.04 to 0.01 (upper three panels in Figure 5). A similar dynamic was found for the root‐free zone, although chargeabilities were slightly higher than for maize. While sugar beet started out with a similar chargeability to that encountered at the maize locations, it increased significantly towards timestep t2 and t3, with chargeability values of up to 0.07 and 0.1, respectively. Additionally, the chargeability distribution at timesteps t2 and t3 was much wider than for the maize plants.

Since we observed the development of a low‐frequency peak in the sugar beet data (Figure 4), we additionally estimated the mean relaxation time for lower frequencies by using a Debye decomposition on the sugar beet spectra up to a frequency of 55 Hz. The results (Figure 5d) show that the mean low‐frequency relaxation time near the sugar beet plants started at average values of 17 ms at timestep t1, and increased significantly at timesteps t2 and t3 (to average values of 50 and 80 ms, respectively). This trend corresponded to the shift of the polarization peak towards lower frequencies, as shown in Figure 4, and is consistent with the inverse relationship between relaxation time and peak frequency (e.g., Weigand and Kemna 2016). Additionally, the relaxation time distribution became broader at timesteps t2 and t3.

### 3.2 Comparison of Electrical Signatures to Root Validation Data

**Figure A3:**
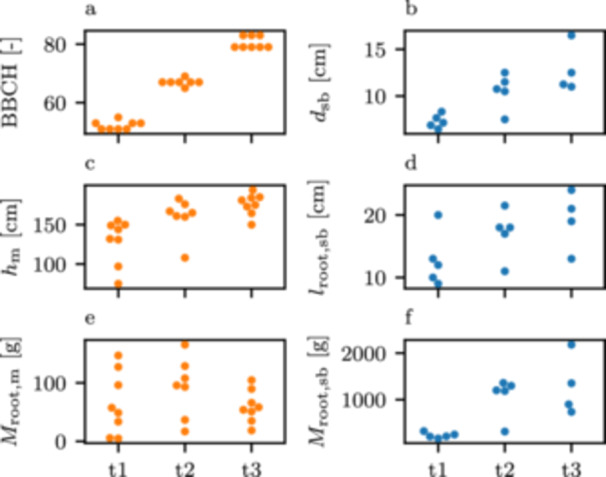
Swarmplots of root traits for all plants within the profiles. Displayed are BBCH index of maize (a), maize plant height (c), maize fresh root biomass (e), maximum sugar beet diameter (b), sugar beet length (d), and sugar beet fresh biomass (f). Maize plant traits are colored in orange, sugar beet traits in blue. [Color figure can be viewed at wileyonlinelibrary.com]

An overview of the root and shoot validation traits collected during the experiment is shown in the Appendix in Figure A3. Maize plant growth stages were estimated to be in the middle of tasseling at timestep t1 (BBCH of 55), end of flowering at timestep t2 (BBCH of 69), and ripening at timestep t3 (BBCH of 83). Maize plant height overall increased for all timesteps. No significant temporal trend was found for the maize root biomass. For sugar beet, maximum beet diameter, beet length, and fresh beet biomass increased during the season, with the largest growth occuring between timesteps t1 and t2. An analysis of variance revealed that temporal trends in all parameters, except maize root biomass, were statistically significant. This was due to high variance in maize root biomass for each individual timestep, and suggests that temporal variability in this parameter should be interpreted with caution.

**Figure 6:**
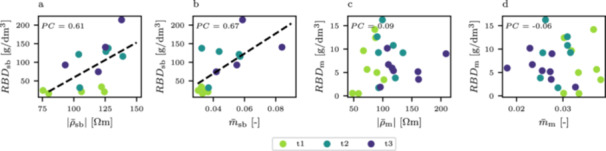
Comparison of the average complex resistivity magnitude ($\left|\overset{-}{\rho }\right|$) and chargeability ($\overset{-}{m}$) with the root biomass densitity near the sugar beet plants (a, b) and maize plants (c, d), respectively. PC denotes the Pearson correlation coefficient, dashed black lines show the best linear fit between parameters. [Color figure can be viewed at wileyonlinelibrary.com]

We compared the average complex resistivity magnitude and chargeability near the sugar beet and maize plants with the root biomass density for all timesteps. While there was a weak correlation between complex resistivity magnitude and root biomass density for the sugar beet plants (with a pearson correlation coefficient (PC) of 0.61 and *p* value of 0.01), no significant correlation was found for the maize plants (Figure 6a and c). Similarly, there was an agreement between chargeability and root biomass density for sugar beet (PC = 0.67, *p* value = 0.005), while maize did not show any correlation (Figure 6b and d).

**Figure 7:**
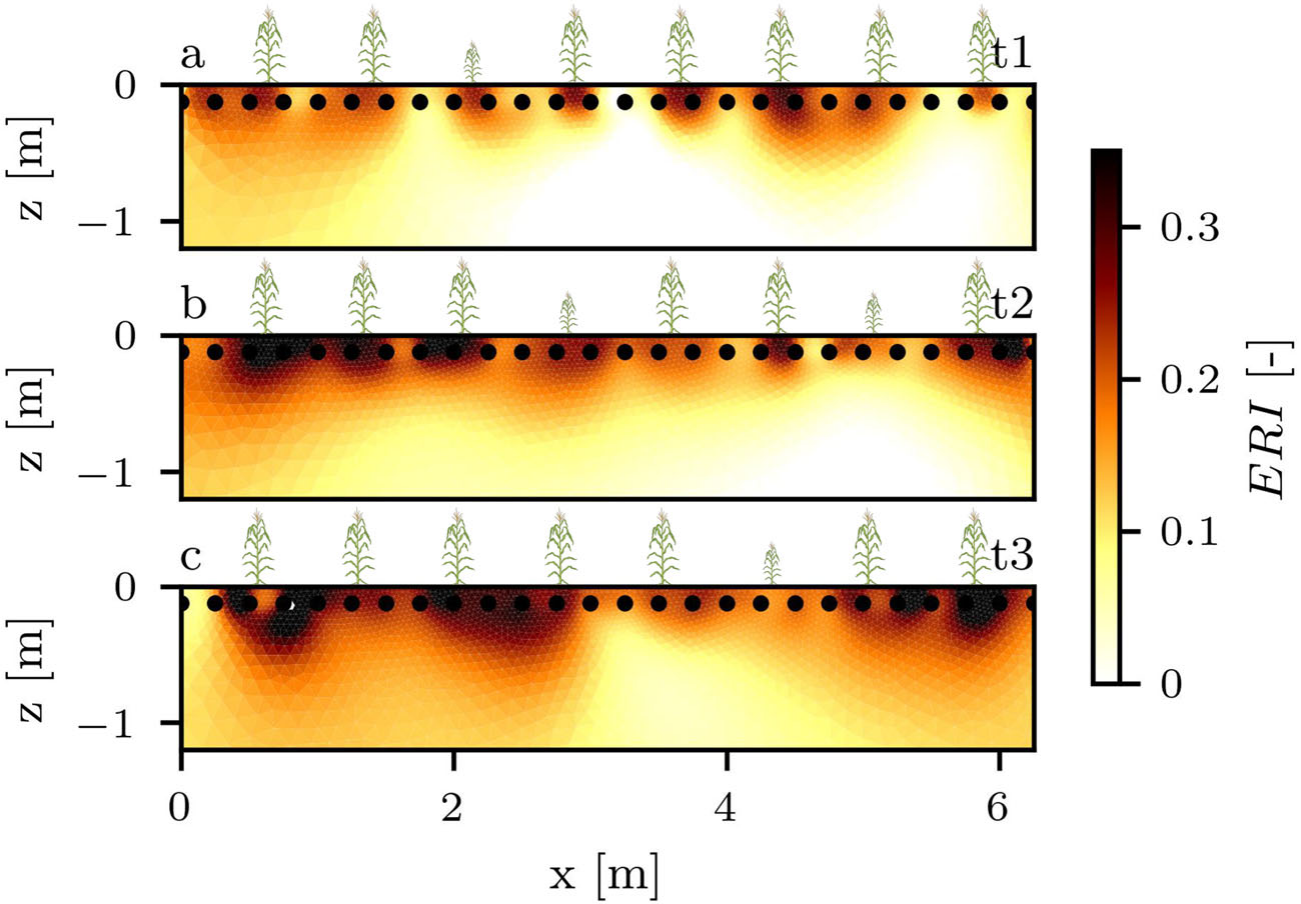
ERI imaging results of the maize plant section for all three timesteps. Plant icons mark the plant locations in the profiles. Smaller icons indicate an off‐centered plant location (in direction perpendicular to the image plane). [Color figure can be viewed at wileyonlinelibrary.com]

**Figure 8:**
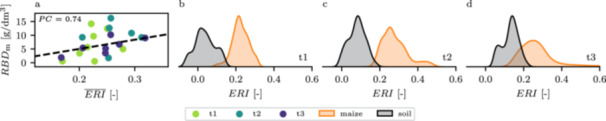
(a) Average ERI values at the maize plant locations, plotted against the root biomass density. PC denotes the Pearson correlation coefficient. Plots b. to d. show the distribution of ERI values at the maize plant locations (orange) and soil (black) for timesteps t1, t2 and t3, respectively. [Color figure can be viewed at wileyonlinelibrary.com]

For the maize plant area, we additionally computed the ERI from the imaginary part of the complex conductivity at 1 kHz and 3.125 Hz according to Section 2.3. As stated previously, due to normalization with the (low‐frequency) soil polarization response, the ERI serves as a proxy for root presence. The tomographic results (Figure 7) show an increased ERI near the plant positions at all timesteps, with values increasing over time. The anomalies progressed towards lower soil layers over time, and were spatially most defined at timestep t1. We found a correlation between the average ERI at the maize plant positions and the root biomass density (PC = 0.74, *p*‐value = 0.027; see Figure 8a). Two datapoints severely deviated from the bulk of retrieved ERI values (the first plant in profile t2 with an average ERI of 0.41, and the last plant in profile t3 with an average ERI of 0.5). The elevated ERIs for these plants were caused by imaginary parts close to zero at 3.125 Hz, and were treated as outliers and excluded from the analysis shown in Figure 8a. To investigate its variability in rooted and root‐free soil zones, we extracted the ERI at the maize plant locations, as well as all grid cells in a depth from 50 to 100 cm, which we classified as mostly root‐free (see Section 2.4.2). The distributions of the ERI (Figure 8b‐d) show that at the plant locations, maximum values increased over time, while the general shape of the distribution flattened towards later timesteps. The ERI values of cells classified as soil did not show a significant change over the season, and were generally lower than values of cells near the maize plants.

## 4 Discussion

In our results, we showed that, with appropriate data error estimates and correction procedures for inductive effects, complex resistivity imaging of plant root systems with frequencies of up to 1 kHz is achievable, and the inferred electrical parameters can be used to non‐invasively estimate root traits in field conditions. Although the application of sEIT is more challenging and time‐consuming than ERT, the additional information of polarization processes can improve estimation of root traits, and reduce uncertainty due to environmental factors like soil saturation and temperature. The following sections address the specific workflow and associated experimental challenges of this study, provide an interpretation of the results, and propose future directions for electrical field phenotyping trials.

### 4.1 Experimental Procedure

To begin, we want to address the reliability of root validation data collected in this study. While plant trait trends over time are evident (Figure A3), the shovelomics method used is prone to errors due to its destructive and incomplete sampling procedure. In particular, maize root biomass estimation is likely affected by the loss of fine root material during excavation and washing (Bucksch et al. 2014). The rapid in‐field sampling of root traits, while efficient, therefore comes at the cost of high uncertainty in biomass estimates. We did not observe a temporal trend in the maize root biomass data. Because of the limited sampling depth of 25 cm, a trend is not necessarily expected, as most root growth after tasseling happens in deeper soil layers as the top soil dries out (Sharp and Davies 1985). However, at this point, we cannot say if the low sampling depth, or suspected inaccuracy of the shovelomics method led to this result. For future studies aiming to establish quantitative relationships between electrical measurements and root traits, we recommend employing more precise validation methods, such as root coring (e.g., Burridge et al. 2020) or minirhizotron facilities (e.g., Lärm et al. 2023), and increasing the number of replicates. These improvements would not only yield more reliable biomass estimates, but also facilitate correlating electrical parameters with root traits in deeper soil layers.

In our electrical imaging results, some areas near the surface exhibited slightly positive resistivity phase values at lower frequencies (Figure 3). While Tsukanov and Schwartz (2023) reported that the presence of roots may lower the overall polarization of the combined soil‐root volume, which could have caused this phenomenon, the positive phase value areas are not confined to the plant positions. We therefore believe that a more likely explanation are polarization effects caused by the used stainless steel electrodes, typically occurring at frequencies below 100 Hz. While practical in application, Zimmermann and Huisman (2024) recently reported that when using stainless steel rods as potential electrodes in field sEIT measurements, significant errors in the retrieved phase readings for γ‐type electrode configurations, as partially used in our study, may occur. For future field studies on root systems, especially when lower measurement frequencies are of importance (e.g. to compute the ERI), these configurations should therefore be avoided to prevent electrode polarization effects. Alternatively, nonpolarizable electrodes may be used (e.g., Zimmermann and Huisman 2024). We also noted a positive phase anomaly below the sugar beet plants at timestep t2 and t3. Strong resistivity contrasts near electrodes have been shown to cause artifacts in electrical imaging applications (e.g., Wilkinson et al. 2008). Thus, we attribute the positive phase values to the strong contrast in electrical properties between sugar beet and soil, as well as to the smoothing process inherent in the inversion.

### 4.2 Resistivity Dynamics

We found that the resistivity magnitude at the sugar beet locations correlates with the root biomass density of the beets, while resistivity in the maize plots could not be used to estimate root traits of the respective plants (Figure 6a and c). We believe that this is due to the sugar beet size, which significantly influences the conduction properties, and therefore the retrieved resistivity distribution, of the medium. This may also explain the (in comparison to maize) initially higher resistivity levels near the sugar beet plants (Figure 2), and is in line with other studies that use electrical measurements on large biological targets, for example tree roots (e.g., Amato et al. 2008; Rossi et al. 2011). In contrast, the maize root density in our experiment was likely too low to cause a significant resistivity increase through biomass itself. Notably, even among sugar beet plants, there is considerable resistivity variability within each timestep that is not reflected in biomass variability (Figure 6a). We attribute this partly to soil heterogeneity and water content variations, as well as spatial variations in the sensitivity of the measurement scheme. Although a correlation across all timesteps is possible, analyzing individual timesteps was not feasible due to this variability, and the limited number of samples.

While some studies could establish relationships between resistivity and root biomass for fine root systems with higher root densities (e.g., Amato et al. 2009), this dependency is more often caused by the sensitivity of electrical methods to water content dynamics in the soil (e.g., Srayeddin and Doussan 2009; Garré et al. 2011; Chou et al. 2024). Sharp and Davies (1985) stated that under high soil moisture conditions, the majority of maize root biomass and water uptake is concentrated in the upper 50 cm of soil, which corresponds to the observed resistivity increase in the shallow soil areas in the tomograms (Figure 2). Notably, resistivity increases more significantly near the maize plants than near the sugar beet plants over time. We believe this could be due to (a) higher root water uptake by maize over the season (e.g., Gerbens‐Leenes and Hoekstra 2012) and (b) the canopy of the sugar beet plants, which shields nearby soil from radiation (Brown et al. 1987), thereby reducing soil water evaporation. Interestingly, we found that, although resistivity increases for all timesteps near maize, no such trend is visible in the root biomass data (Figure A3e). Keeping the mentioned limitations of the shovelomics method in mind, we have two explanations for this: First, it is possible that although resistivity increased through evaporation, no significant shallow root growth happened between timestep t2 and t3 (see Section 4.1). Secondly, the onset of root senescence (Chen et al. 2015; Sha et al. 2023) could have reduced the overall fresh root biomass in the last timestep, thus causing a mismatch between resistivity and biomass. This result highlights that, although resistivity may serve as a proxy for fine root biomass under favorable conditions, significant variability in soil water content or physiological decay of the root system may hinder accurate biomass estimation via resistivity measurements alone.

### 4.3 Polarizability and ERI

When using the polarization strength (i.e., total chargeability) as an indicator for root presence, we observed a (compared to resistivity magnitude) slightly better correlation with the root biomass density (Figure 6b) at the sugar beet locations. Similar to the resistivity, there exists some variability in chargeability within each timestep that inhibits the analysis of individual timesteps. It is noteworthy that high variability of data is most prominent in timestep t2. We believe that this variability stems from the observed inversion artifact at 1 Hz near the sugar beet plants (Figure 3), and removing the two sugar beet plants that are closest to the positive phase anomaly from the analysis improves the overall correleation to a PC of 0.81. This result shows that chargeability can be a promising parameter for biomass estimation of larger roots, especially when measurement design choices are implemented that have the capability to avoid inversion artifacts (see Section 4.1). However, if the survey design is not chosen carefully, inversion artifacts or heterogeneous sensitivity patterns may hinder the application in high‐throughput phenotyping studies (e.g, Bai et al. 2019), and more research is needed to validate the method for large‐scale field trials.

No dependency between chargeability and root biomass density could be found near the maize plants (Figure 6d). As stated above, an explanation for this observation is the difference in present biomass for sugar beet and maize, as well as the superimposed polarization signature of the soil, effectively masking the root signature at the maize plant locations. It is noteworthy that the chargeability near the maize plants is inversely correlated with the resistivity (and thus, water saturation) dynamics, a behavior commonly found in induced polarization measurements of porous media samples (e.g., Breede et al. 2012; Gao et al. 2019), and also evident in the soil chargeability in Figure 5a‐c. It supports the hypothesis that total polarization strength in the maize rooting area is dominated by the soil signature. In contrast, the chargeability at the sugar beet locations increases despite the rising resistivity, indicating that the root itself dominates the polarization signature in this case.

The introduced ERI seems to overcome the above mentioned soil masking effect for the maize root areas by normalizing the high‐frequency (combined root and soil) with the low‐frequency (soil‐dominated) polarization signature, achieving a significant correlation between ERI and root biomass density of the maize plants (Figure 8a). Since soil water content variations affect both the high‐ and low‐frequency polarization (e.g., Jougnot et al. 2010; Revil et al. 2023), we believe that the normalization on the low‐frequency polarization effectively dampens the soil water content influence in the high‐frequency polarization, enabling the identification of root polarization present at this frequency. We are aware that the assumption of soil water content influencing all frequencies similarly (and linearly) is likely not correct for all soils, and in the best case, a laboratory calibration measurement of soil from the experimental site could be used to better estimate the influence of variable soil moisture on the ERI. Still, previous research has shown that root matter exhibits a strong high‐frequency polarization signature (e.g., Weigand and Kemna 2017; Ehosioke et al. 2023; Michels et al. 2024), suggesting that variations due to inaccurate soil water content normalization are likely small compared to the impact of root biomass on the ERI. In this study, the ERI of the soil signature was computed from deeper soil layers that did not exhibit strong variations in soil water content. Further research towards the variability of the ERI with regard to more severe fluctuations of water content, salinity, and temperature are needed to fully assess its usability for root trait estimation in dynamic conditions. Additionally, we note that the ERI is a site‐specific quantity, as different rooting media naturally result in different spectral polarization behavior. It may therefore be difficult to compare measurements from different experimental sites when no baseline measurement of the soil is performed. Consequentially, the true strength of the ERI could be an application in monitoring experiments over a whole season, where the first (root‐less) timestep is used as a reference measurement to calculate changes in the ERI, thus making it comparable to other measurement sites independent of the background polarization.

While not the main reason for its application, we found that a methodological advantage of the ERI is the robustness of imaging results towards deeper soil layers. Electrical surveys typically lose resolution with depth due to the sensitivity patterns of employed four‐point measurements, which diminish in deeper layers farther away from the electrodes (e.g., Furman et al. 2003). By division of high‐ and low‐frequency imaginary conductivities, which are both similarly affected by the imposed sensitivity pattern of the measurement design, the perceived loss of contrast with depth is reduced (e.g., Weigand et al. 2017), enabling a vertically consistent and more robust analysis of root traits. The ERI approach may therefore be especially viable when sensitivity of the electrical survey is limited by sparse electrode configurations, or for measurement schemes that are expected to exhibit considerable sensitivity variations. Since our study focused on validation data from the upper 25 cm of soil, future research should assess the reliability of the ERI for root biomass estimation in deeper soil layers.

### 4.4 Polarization Length Scales

**Figure 9:**
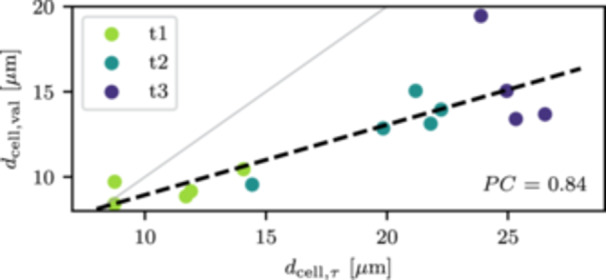
Storage root cell diameter derived from electrical measurements ($d_{\text{cell},\tau }$), plotted against estimated validation storage cell diameter ($d_{\text{cell},\text{val}}$) that was derived from the literature scaling relationship. PC denotes the Pearson correlation coefficient. The dashed black line indicates the best linear data fit, the grey line the 1:1 relation between both parameters. [Color figure can be viewed at wileyonlinelibrary.com]

Another important result of this study is the development of the (besides the high‐frequency polarization) low‐frequency polarization peak at the sugar beet positions, corresponding to the increase of low‐frequency relaxation times over the season (see Figure 4 and lower panel of Figure 5). We hypothesize that this bimodality in polarization behaviour is caused by the development of differently sized vascular tissue cells and storage parenchyma in sugar beet (e.g., Zamski and Azenkot 1981). A convincing argument for this idea is that the storage cells are similar in size to the vascular tissue cells during early growth stages (corresponding to timestep t1), and only later in the season significantly increase their diameter (e.g., Milford 1973). The development of the low‐frequency polarization peak may therefore be caused by development of the larger storage cells. Since maize does not develop storage cells, it is logical that no pronounced low‐frequency peak is observed in its polarization response (Figure 4). We used Eq. 9 and the mean relaxation times obtained from the low‐frequency Debye decomposition to estimate the low‐frequency polarization length scale in the sugar beet plants, hypothetically corresponding to the storage parenchyma diameter. Assuming a typical diffusion coefficient of 10^–9^ m^2^/s (e.g., Weigand and Kemna 2019; Tsukanov and Schwartz 2020), the mean relaxation times translate to average cell diameters of 11 μm at timestep t1, 20 μm at timestep t2, and 25 μm at timestep t3. These results are in agreement with studies investigating vascular and storage cell sizes of sugar beet (e.g., Milford 1973; Zamski and Azenkot 1981; Nause et al. 2023). Unfortunately, no measurement of cell diameter was performed during the experiment to confirm this hypothesis. Instead, using the data of Hoffmann (2010) that suggest a linear relationship between macroscopic beet diameter and storage cell diameter, we estimated the mean storage cell diameter in our experiment using the recorded beet diameter. We found a correlation of estimated validation ($d_{\text{cell},\text{val}}$) and electrically derived ($d_{\text{cell},\tau }$) storage cell diameter (Figure 9, PC of 0.84 and p‐value of $8\cdot 10^{-5}$). However, the electrically derived cell diameter is overestimated for timesteps t2 and t3. At this point, we can not say if the misfit is stemming from an overestimation of the electrically derived parameter, or an underestimation of the validation root trait, which was computed from an empirical relationship that might not align with the specific conditions of our experiment. Nevertheless, our results suggest that sEIT is sensitive to the internal microscopic structures of root systems, making it a promising tool for characterizing root cell size. Despite using a macroscopic measurement setup (with associated macroscopic spatial imaging resolution), the frequency dependence of the polarization signature enables inference of information at much smaller scales. Further research should focus on establishing relationships between cell diameter and relaxation time across different root systems.

## 5 Conclusions

In this study, we used spectral electrical impedance tomography as a tool for in‐situ root phenotyping of sugar beet and maize. To address our research question of whether root polarization signatures can be measured at the field scale, we successfully conducted field‐scale spectroscopic impedance measurements across a frequency range of 0.1 Hz to 1 kHz. Consistent inversion results were achieved through employment of inductive correction procedures and data filtering schemes. Our complex resistivity imaging results revealed an increase of polarization for both plant types at higher measurement frequencies. Additionally, sugar beet developed a low‐frequency polarization peak, which shifted to lower frequencies as the season progressed. To assess whether root and soil polarization signatures can be differentiated (research question two) and whether root traits can be extracted from the polarization signature (research question three), we performed a Debye decomposition to extract the total chargeability and mean relaxation time for all grid cells. While resistivity magnitude and chargeability of sugar beet were correlated with root biomass density, the background soil signature inhibited the analysis of maize imaging results in a similar way. We attribute this to the low biomass density of maize, compared to sugar beet, and the dynamic changes in soil water content affecting background soil polarization. We introduced the electrical root index ERI as a proxy for root presence with mitigated sensitivity to water content variations, and demonstrated its correlation with the root biomass density of the maize plants. Furthermore, we related the shift in low‐frequency relaxation times near the sugar beet plants to the development of larger storage parenchyma, and demonstrated this link by correlating electrically derived polarization length scales with storage cell diameters. Overall, our findings highlight the potential of sEIT for the in‐situ characterization of macro‐ and microscopic root system traits. Further sEIT studies targeting root systems at the field scale should focus on the calibration of soil water content‐induced variations in the polarization signal and assessing the robustness of the ERI against these changes. Additionally, future research should explore the link between microscopic structural features in root systems and the relaxation time distributions of different plant types.

## Conflicts of Interest

The authors declare no conflicts of interest.

## Data Availability

The data that support the findings of this study are openly available in Zenodo at https://doi.org/10.5281/zenodo.14780503.

## References

[pce70049-bib-0001] Amato, M. , B. Basso , G. Celano , G. Bitella , G. Morelli , and R. Rossi . 2008. “In Situ Detection of Tree Root Distribution and Biomass by Multi‐ Electrode Resistivity Imaging.” Tree Physiology 28: 1441–1448. 10.1093/treephys/28.10.1441. 18708325

[pce70049-bib-0002] Amato, M. , G. Bitella , R. Rossi , J. A. Gómez , S. Lovelli , and J. J. F. Gomes . 2009. “Multi‐Electrode 3D Resistivity Imaging of Alfalfa Root Zone.” European Journal of Agronomy 31, no. 4: 213–222. 10.1016/j.eja.2009.08.005.

[pce70049-bib-0003] Bagnall, G. C. , S. A. Altobelli , M. S. Conradi , et al. 2022. “Design and Demonstration of a Low‐Field Magnetic Resonance Imaging Rhizotron for in‐Field Imaging of Energy Sorghum Roots.” Plant Phenome Journal 5, no. 1: e20038. 10.1002/ppj2.20038.

[pce70049-bib-0004] Bai, G. , Y. Ge , D. Scoby , et al. 2019. “NU‐Spidercam: A Large‐Scale, Cable‐Driven, Integrated Sensing and Robotic System for Advanced Phenotyping, Remote Sensing, and Agronomic Research.” Computers and Electronics in Agriculture 160: 71–81. 10.1016/j.compag.2019.03.009.

[pce70049-bib-0005] Bairlein, K. , M. Bücker , A. Hördt , and B. Hinze . 2016. “Temperature Dependence of Spectral Induced Polarization Data: Experimental Results and Membrane Polarization Theory.” Geophysical Journal International 205, no. 1: 440–453. 10.1093/gji/ggw027.

[pce70049-bib-0006] Binley, A. , and L. Slater . 2020. Resistivity and Induced Polarization: Theory and Applications to the Near‐Surface Earth. Cambridge University Press.

[pce70049-bib-0007] Binley, A. , L. D. Slater , M. Fukes , and G. Cassiani . 2005. “Relationship Between Spectral Induced Polarization and Hydraulic Properties of Saturated and Unsaturated Sandstone.” Water Resources Research 41, no. 12: 2005WR004202. 10.1029/2005WR004202.

[pce70049-bib-0008] Blanchy, G. , W. Deroo , T. De Swaef , et al. 2025. “Closing the Phenotyping Gap With non‐Invasive Belowground Field Phenotyping.” SOIL 11, no. 1: 67–84. 10.5194/soil-11-67-2025.

[pce70049-bib-0009] Blanchy, G. , N. Virlet , P. Sadeghi‐Tehran , et al. 2020. “Time‐Intensive Geoelectrical Monitoring Under Winter Wheat.” Near Surface Geophysics 18, no. 4: 413–425. 10.1002/nsg.12107.

[pce70049-bib-0010] Bogena, H. R. , M. Herbst , J. A. Huisman , U. Rosenbaum , A. Weuthen , and H. Vereecken . 2010. “Potential of Wireless Sensor Networks for Measuring Soil Water Content Variability.” Vadose Zone Journal 9, no. 4: 1002–1013. 10.2136/vzj2009.0173.

[pce70049-bib-0011] Breede, K. , A. Kemna , O. Esser , E. Zimmermann , H. Vereecken , and J. A. Huisman . 2012. “Spectral Induced Polarization Measurements on Variably Saturated Sand‐Clay Mixtures.” Near Surface Geophysics 10, no. 6: 479–489. 10.3997/1873-0604.2012048.

[pce70049-bib-0012] Brown, K. F. , A. B. Messem , R. J. Dunham , and P. V. Biscoe . 1987. “Effect of Drought on Growth and Water use of Sugar Beet.” Journal of Agricultural Science 109, no. 3: 421–435. 10.1017/S0021859600081636.

[pce70049-bib-0013] Bücker, M. , A. Flores Orozco , S. Undorf , and A. Kemna . 2019. “On the Role of Stern‐ and Diffuse‐Layer Polarization Mechanisms in Porous Media.” Journal of Geophysical Research: Solid Earth 124, no. 6: 5656–5677. 10.1029/2019JB017679.

[pce70049-bib-0014] Bucksch, A. , J. Burridge , L. M. York , et al. 2014. “Image‐Based High‐Throughput Field Phenotyping of Crop Roots.” Plant Physiology 166, no. 2: 470–486. 10.1104/pp.114.243519. 25187526 PMC4213080

[pce70049-bib-0015] Burridge, J. D. , C. K. Black , E. A. Nord , et al. 2020. “An Analysis of Soil Coring Strategies to Estimate Root Depth in Maize (*Zea mays*) and Common Bean (*Phaseolus vulgaris*).” Plant Phenomics 2020: 2020/3252703. 10.34133/2020/3252703. PMC7706327 33313549

[pce70049-bib-0016] Carrera, A. , M. Longo , I. Piccoli , B. Mary , G. Cassiani , and F. Morari . 2022. “Electro‐Magnetic Geophysical Dynamics Under Conservation and Conventional Farming.” Remote Sensing 14, no. 24: 6243. 10.3390/rs14246243.

[pce70049-bib-0017] Carrera, A. , L. Peruzzo , M. Longo , G. Cassiani , and F. Morari . 2024. “Uncovering Soil Compaction: Performance of Electrical and Electromagnetic Geophysical Methods.” SOIL 10, no. 2: 843–857. 10.5194/soil-10-843-2024.

[pce70049-bib-0018] Chen, Y. , J. Zhang , Q. Li , et al. 2015. “Effects of Nitrogen Application on Post‐Silking Root Senescence and Yield of Maize.” Agronomy Journal 107, no. 3: 835–842. 10.2134/agronj14.0509.

[pce70049-bib-0019] Chimungu, J. G. , K. M. Brown , and J. P. Lynch . 2014. “Large Root Cortical Cell Size Improves Drought Tolerance in Maize.” Plant Physiology 166, no. 4: 2166–2178. 10.1104/pp.114.250449. 25293960 PMC4256844

[pce70049-bib-0020] Chloupek, O. 1972. “The Relationship Between Electric Capacitance and Some Other Parameters of Plant Roots.” Biologia Plantarum 14, no. 3: 227–230. 10.1007/BF02921255.

[pce70049-bib-0021] Chou, C. , L. Peruzzo , N. Falco , et al. 2024. “Improving Evapotranspiration Computation With Electrical Resistivity Tomography in a Maize Field.” Vadose Zone Journal 23, no. 1: e20290. 10.1002/vzj2.20290.

[pce70049-bib-0022] Cimpoiaşu, M. O. , O. Kuras , T. Pridmore , and S. J. Mooney . 2020. “Potential of Geoelectrical Methods to Monitor Root Zone Processes and Structure: A Review.” Geoderma 365: 114232. 10.1016/j.geoderma.2020.114232.

[pce70049-bib-0023] Cseresnyés, I. , A. Füzy , S. Kabos , B. Kelemen , K. Rajkai , and T. Takács . 2024a. “Monitoring of Plant Water Uptake by Measuring Root Dielectric Properties on a Fine Timescale: Diurnal Changes and Response to Leaf Excision.” Plant Methods 20, no. 1: 5. 10.1186/s13007-023-01133-8. 38195647 PMC10775601

[pce70049-bib-0024] Cseresnyés, I. , B. Kelemen , T. Takács , et al. 2021. “Electrical Capacitance Versus Minirhizotron Technique: A Study of Root Dynamics in Wheat–Pea Intercrops.” Plants 10, no. 10: 1991. 10.3390/plants10101991. 34685800 PMC8540429

[pce70049-bib-0025] Cseresnyés, I. , K. Szitár , K. Rajkai , et al. 2018. “Application of Electrical Capacitance Method for Prediction of Plant Root Mass and Activity in Field‐Grown Crops.” Frontiers in Plant Science 9: 93. 10.3389/fpls.2018.00093. 29449861 PMC5799269

[pce70049-bib-0026] Cseresnyés, I. , T. Takács , and A. Füzy . 2024b. “Detection of Plant Cadmium Toxicity by Monitoring Dielectric Response of Intact Root Systems on a Fine Timescale.” Environmental Science and Pollution Research 31: 30555–30568. 10.1007/s11356-024-33279-w. 38607480 PMC11096224

[pce70049-bib-0027] Dahlin, T. 2000. “Short Note on Electrode Charge‐up Effects in DC Resistivity Data Acquisition Using Multi‐Electrode Arrays.” Geophysical Prospecting 48, no. 1: 181–187. 10.1046/j.1365-2478.2000.00172.x.

[pce70049-bib-0028] Dalton, F. N. 1995. “In‐Situ Root Extent Measurements by Electrical Capacitance Methods.” Plant and Soil 173, no. 1: 157–165. 10.1007/BF00155527.

[pce70049-bib-0029] Ehosioke, S. , S. Garré , J. A. Huisman , et al. 2023. “Spectroscopic Approach Toward Unraveling the Electrical Signature of Roots.” Journal of Geophysical Research: Biogeosciences 128, no. 4: e2022JG007281. 10.1029/2022JG007281.

[pce70049-bib-0030] Ehosioke, S. , F. Nguyen , S. Rao , et al. 2020. “Sensing the Electrical Properties of Roots: A Review.” Vadose Zone Journal 19, no. 1: e20082. 10.1002/vzj2.20082.

[pce70049-bib-0031] Ellis, T. W. , W. Murray , K. Paul , et al. 2013. “Electrical Capacitance as a Rapid and non‐Invasive Indicator of Root Length.” Tree Physiology 33, no. 1: 3–17. 10.1093/treephys/tps115. 23243029

[pce70049-bib-0032] Falk, K. G. , T. Z. Jubery , S. V. Mirnezami , et al. 2020. “Computer Vision and Machine Learning Enabled Soybean Root Phenotyping Pipeline.” Plant Methods 16, no. 1: 5. 10.1186/s13007-019-0550-5. 31993072 PMC6977263

[pce70049-bib-0033] Flores Orozco, A. , L. Aigner , and J. Gallistl . 2021. “Investigation of Cable Effects in Spectral Induced Polarization Imaging at the Field Scale Using Multicore and Coaxial Cables.” Geophysics 86, no. 1: E59–E75. 10.1190/geo2019-0552.1.

[pce70049-bib-0034] Furman, A. , T. P. A. Ferré , and A. W. Warrick . 2003. “A Sensitivity Analysis of Electrical Resistivity Tomography Array Types Using Analytical Element Modeling.” Vadose Zone Journal 2, no. 3: 416–423. 10.2136/vzj2003.4160.

[pce70049-bib-0035] Gao, H. , J. Zhang , C. Liu , et al. 2019. “Efficient Bayesian Inverse Modeling of Water Infiltration in Layered Soils.” Vadose Zone Journal 18, no. 1: 1–13. 10.2136/vzj2018.12.0213.

[pce70049-bib-0036] Garré, S. , T. Günther , J. Diels , and J. Vanderborght . 2012. “Evaluating Experimental Design of ERT for Soil Moisture Monitoring in Contour Hedgerow Intercropping Systems.” Vadose Zone Journal 11, no. 4: vzj2011.0186. 10.2136/vzj2011.0186.

[pce70049-bib-0037] Garré, S. , M. Javaux , J. Vanderborght , L. Pagès , and H. Vereecken . 2011. “Three‐Dimensional Electrical Resistivity Tomography to Monitor Root Zone Water Dynamics.” Vadose Zone Journal 10, no. 1: 412–424. 10.2136/vzj2010.0079.

[pce70049-bib-0038] Gerbens‐Leenes, W. , and A. Y. Hoekstra . 2012. “The Water Footprint of Sweeteners and Bio‐Ethanol.” Environment International 40: 202–211. 10.1016/j.envint.2011.06.006. 21802146

[pce70049-bib-0039] Gioia, T. , A. Galinski , H. Lenz , et al. 2017. “GrowScreen‐PaGe, a Non‐Invasive, High‐Throughput Phenotyping System Based on Germination Paper to Quantify Crop Phenotypic Diversity and Plasticity of Root Traits Under Varying Nutrient Supply.” Functional Plant Biology 44, no. 1: 76. 10.1071/FP16128. 32480548

[pce70049-bib-0040] Gu, H. , I. Cseresnyés , J. R. Butnor , et al. 2024. “Advancing Noninvasive and Nondestructive Root Phenotyping Techniques: A Two‐Phase Permittivity Model for Accurate Estimation of Root Volume.” Geoderma 442: 116773. 10.1016/j.geoderma.2024.116773.

[pce70049-bib-0041] Herrero‐Huerta, M. , V. Meline , A. S. Iyer‐Pascuzzi , A. M. Souza , M. R. Tuinstra , and Y. Yang . 2021. “4D Structural Root Architecture Modeling From Digital Twins by X‐Ray Computed Tomography.” Plant Methods 17, no. 1: 123. 10.1186/s13007-021-00819-1. 34863243 PMC8642944

[pce70049-bib-0042] Hoffmann, C. M. 2010. “Sucrose Accumulation in Sugar Beet Under Drought Stress.” Journal of Agronomy and Crop Science 196, no. 4: 243–252. 10.1111/j.1439-037X.2009.00415.x.

[pce70049-bib-0043] Jougnot, D. , A. Ghorbani , A. Revil , P. Leroy , and P. Cosenza . 2010. “Spectral Induced Polarization of Partially Saturated Clay‐Rocks: A Mechanistic Approach.” Geophysical Journal International 180, no. 1: 210–224. 10.1111/j.1365-246X.2009.04426.x.

[pce70049-bib-0044] Kelter, M. , J. A. Huisman , E. Zimmermann , and H. Vereecken . 2018. “Field Evaluation of Broadband Spectral Electrical Imaging for Soil and Aquifer Characterization.” Journal of Applied Geophysics 159: 484–496. 10.1016/j.jappgeo.2018.09.029.

[pce70049-bib-0045] Kemna, A. 2000. Tomographic inversion of complex resistivity—theory and application (Ph.D. thesis). Ruhr‐Universität Bochum.

[pce70049-bib-0046] Kendall, W. A. , G. A. Pederson , and R. R. Hill . 1982. “Root Size Estimates of Red Clover and Alfalfa Based on Electrical Capacitance and Root Diameter Measurements.” Grass and Forage Science 37, no. 3: 253–256. 10.1111/j.1365-2494.1982.tb01604.x.

[pce70049-bib-0047] Kessouri, P. , A. Furman , J. A. Huisman , et al. 2019. “Induced Polarization Applied to Biogeophysics: Recent Advances and Future Prospects.” Near Surface Geophysics 17, no. 6: 595–621. 10.1002/nsg.12072.

[pce70049-bib-0048] LaBrecque, D. J. , M. Miletto , W. Daily , A. Ramirez , and E. Owen . 1996. “The Effects of Noise on Occam's Inversion of Resistivity Tomography Data.” Geophysics 61, no. 2: 538–548. 10.1190/1.1443980.

[pce70049-bib-0049] Lärm, L. , F. M. Bauer , and N. Hermes , et al. 2023. “Multi‐Year Belowground Data of Minirhizotron Facilities in Selhausen.” Scientific Data 10, no. 1: 672. 10.1038/s41597-023-02570-9. 37789016 PMC10547842

[pce70049-bib-0050] Liu, S. , N. Begum , T. An , et al. 2021. “Characterization of Root System Architecture Traits in Diverse Soybean Genotypes Using a Semi‐Hydroponic System.” Plants 10, no. 12: 2781. 10.3390/plants10122781. 34961252 PMC8707277

[pce70049-bib-0051] Ludwig, P. , F. Ehmele , M. J. Franca , et al. 2023. “A Multi‐Disciplinary Analysis of the Exceptional Flood Event of July 2021 in Central Europe – Part 2: Historical Context and Relation to Climate Change.” Natural Hazards and Earth System Sciences 23, no. 4: 1287–1311. 10.5194/nhess-23-1287-2023.

[pce70049-bib-0052] Lyklema, J. , S. S. Dukhin , and V. N. Shilov . 1983. “The Relaxation of the Double Layer Around Colloidal Particles and the Low‐Frequency Dielectric Dispersion.” Journal of Electroanalytical Chemistry and Interfacial Electrochemistry 143, no. 1/2: 1–21. 10.1016/S0022-0728(83)80251-4.

[pce70049-bib-0053] Lynch, J. 1995. “Root Architecture and Plant Productivity.” Plant Physiology 109, no. 1: 7–13. 10.1104/pp.109.1.7. 12228579 PMC157559

[pce70049-bib-0054] Lynch, J. P. 2007. “Roots of the Second Green Revolution.” Australian Journal of Botany 55, no. 5: 493. 10.1071/BT06118.

[pce70049-bib-0055] Martins, S. M. , G. G. Brito , W. C. Gonçalves , et al. 2020. “Phenoroots: An Inexpensive Non‐Invasive Phenotyping System to Assess the Variability of the Root System Architecture.” Scientia Agricola 77, no. 5: e20180420. 10.1590/1678-992x-2018-0420.

[pce70049-bib-0056] Mary, B. , V. Iván , F. Meggio , et al. 2023. “Imaging of the Electrical Activity in the Root Zone Under Limited‐Water‐Availability Stress: A Laboratory Study for *Vitis vinifera* .” Biogeosciences 20, no. 22: 4625–4650. 10.5194/bg-20-4625-2023.

[pce70049-bib-0057] Mary, B. , L. Peruzzo , J. Boaga , et al. 2020. “Time‐Lapse Monitoring of Root Water Uptake Using Electrical Resistivity Tomography and Mise‐À‐La‐Masse: A Vineyard Infiltration Experiment.” SOIL 6, no. 1: 95–114. 10.5194/soil-6-95-2020.

[pce70049-bib-0058] Meier, U. 2003. “Phenological Growth Stages.” In Phenology: An Integrative Environmental Science, edited by M. D. Schwartz , 269–283. Springer Netherlands.

[pce70049-bib-0059] Michels, V. , C. Chou , M. Weigand , Y. Wu , and A. Kemna . 2024. “Quantitative Phenotyping of Crop Roots With Spectral Electrical Impedance Tomography: A Rhizotron Study With Optimized Measurement Design.” Plant Methods 20, no. 1: 118. 10.1186/s13007-024-01247-7. 39095828 PMC11297745

[pce70049-bib-0060] Milford, G. F. J. 1973. “The Growth and Development of the Storage Root of Sugar Beet.” Annals of Applied Biology 75, no. 3: 427–438. 10.1111/j.1744-7348.1973.tb07991.x.

[pce70049-bib-0061] Nause, N. , F. R. Ispizua Yamati , M. Seidel , A. K. Mahlein , and C. M. Hoffmann . 2023. “Workflow for Phenotyping Sugar Beet Roots by Automated Evaluation of Cell Characteristics and Tissue Arrangement Using Digital Image Processing.” Plant Methods 19, no. 1: 35. 10.1186/s13007-023-01014-0. 37004019 PMC10064576

[pce70049-bib-0062] Nordsiek, S. , and A. Weller . 2008. “A New Approach to Fitting Induced‐Polarization Spectra.” Geophysics 73, no. 6: F235–F245. 10.1190/1.2987412.

[pce70049-bib-0063] Orozco, A. F. , A. Kemna , and E. Zimmermann . 2012. “Data Error Quantification in Spectral Induced Polarization Imaging.” Geophysics 77, no. 3: E227–E237. 10.1190/geo2010-0194.1.

[pce70049-bib-0064] Oswald, S. E. , M. Menon , A. Carminati , P. Vontobel , E. Lehmann , and R. Schulin . 2008. “Quantitative Imaging of Infiltration, Root Growth, and Root Water Uptake via Neutron Radiography.” Vadose Zone Journal 7, no. 3: 1035–1047. 10.2136/vzj2007.0156.

[pce70049-bib-0065] Ozier‐Lafontaine, H. , and T. Bajazet . 2005. “Analysis of Root Growth by Impedance Spectroscopy (EIS).” Plant and Soil 277, no. 1/2: 299–313. 10.1007/s11104-005-7531-3.

[pce70049-bib-0066] Peruzzo, L. , C. Chou , Y. Wu , et al. 2020. “Imaging of Plant Current Pathways for Non‐Invasive Root Phenotyping Using a Newly Developed Electrical Current Source Density Approach.” Plant and Soil 450, no. 1: 567–584. 10.1007/s11104-020-04529-w.

[pce70049-bib-0067] Peruzzo, L. , X. Liu , C. Chou , et al. 2021. “Three‐Channel Electrical Impedance Spectroscopy for Field‐Scale Root Phenotyping.” Plant Phenome Journal 4, no. 1: e20021. 10.1002/ppj2.20021.

[pce70049-bib-0068] Pflugfelder, D. , R. Metzner , D. van Dusschoten , R. Reichel , S. Jahnke , and R. Koller . 2017. “Non‐Invasive Imaging of Plant Roots in Different Soils Using Magnetic Resonance Imaging (MRI).” Plant Methods 13, no. 1: 102. 10.1186/s13007-017-0252-9. 29177002 PMC5693507

[pce70049-bib-0069] Prodan, E. , C. Prodan , and J. H. Miller . 2008. “The Dielectric Response of Spherical Live Cells in Suspension: An Analytic Solution.” Biophysical Journal 95, no. 9: 4174–4182. 10.1529/biophysj.108.137042. 18658215 PMC2567925

[pce70049-bib-0070] Revil, A. , A. Ghorbani , D. Jougnot , and B. Yven . 2023. “Induced Polarization of Clay‐Rich Materials—Part 1: The Effect of Desiccation.” Geophysics 88, no. 4: MR195–MR210. 10.1190/geo2022-0510.1.

[pce70049-bib-0071] Revil, A. , K. Koch , and K. Holliger . 2012. “Is it the Grain Size or the Characteristic Pore Size That Controls the Induced Polarization Relaxation Time of Clean Sands and Sandstones?” Water Resources Research 48, no. 5: 2011WR011561. 10.1029/2011WR011561.

[pce70049-bib-0072] Rossi, R. , M. Amato , G. Bitella , et al. 2011. “Electrical Resistivity Tomography as a Non‐Destructive Method for Mapping Root Biomass in an Orchard.” European Journal of Soil Science 62, no. 2: 206–215. 10.1111/j.1365-2389.2010.01329.x.

[pce70049-bib-0073] Schwarz, G. 1962. “A Theory of the Low‐Frequency Dielectric Dispersion of Colloidal Particles in Electrolyte Solution.” Journal of Physical Chemistry 66, no. 12: 2636–2642. 10.1021/j100818a067.

[pce70049-bib-0074] Sha, Y. , Z. Liu , and Z. Hao , et al. 2023. “Root Growth, Root Senescence and Root System Architecture in Maize Under Conservative Strip Tillage System.” Plant and Soil 495: 253–269. 10.1007/s11104-023-06322-x.

[pce70049-bib-0075] Sharp, R. E. , and W. J. Davies . 1985. “Root Growth and Water Uptake by Maize Plants in Drying Soil.” Journal of Experimental Botany 36, no. 9: 1441–1456. 10.1093/jxb/36.9.1441.

[pce70049-bib-0076] Srayeddin, I. , and C. Doussan . 2009. “Estimation of the Spatial Variability of Root Water Uptake of Maize and Sorghum at the Field Scale by Electrical Resistivity Tomography.” Plant and Soil 319, no. 1–2: 185–207. 10.1007/s11104-008-9860-5.

[pce70049-bib-0077] Středa, T. , J. Haberle , J. Klimešová , et al. 2020. “Field Phenotyping of Plant Roots by Electrical Capacitance—A Standardized Methodological Protocol for Application in Plant Breeding: A Review.” International Agrophysics 34, no. 2: 173–184. 10.31545/intagr/117622.

[pce70049-bib-0078] Teramoto, S. , S. Takayasu , Y. Kitomi , Y. Arai‐Sanoh , T. Tanabata , and Y. Uga . 2020. “High‐Throughput Three‐Dimensional Visualization of Root System Architecture of Rice Using X‐Ray Computed Tomography.” Plant Methods 16, no. 1: 66. 10.1186/s13007-020-00612-6. 32426023 PMC7216661

[pce70049-bib-0079] Teramoto, S. , and Y. Uga . 2020. “A Deep Learning‐Based Phenotypic Analysis of Rice Root Distribution From Field Images.” Plant Phenomics 2020: 2020/3194308. 10.34133/2020/3194308. PMC7706345 33313548

[pce70049-bib-0080] Titov, K. , A. Tarasov , Y. Ilyin , N. Seleznev , and A. Boyd . 2010. “Relationships Between Induced Polarization Relaxation Time and Hydraulic Properties of Sandstone.” Geophysical Journal International 180, no. 3: 1095–1106. 10.1111/j.1365-246X.2009.04465.x.

[pce70049-bib-0081] Trachsel, S. , S. M. Kaeppler , K. M. Brown , and J. P. Lynch . 2011. “Shovelomics: High Throughput Phenotyping of Maize (*Zea mays* L.) Root Architecture in the Field.” Plant and Soil 341, no. 1–2: 75–87. 10.1007/s11104-010-0623-8.

[pce70049-bib-0082] Tsukanov, K. , and N. Schwartz . 2020. “Relationship Between Wheat Root Properties and its Electrical Signature Using the Spectral Induced Polarization Method.” Vadose Zone Journal 19, no. 1: e20014. 10.1002/vzj2.20014.

[pce70049-bib-0083] Tsukanov, K. , and N. Schwartz . 2023. “The Influence of Roots on Soil's Electrical Signature.” Rhizosphere 25: 100670. 10.1016/j.rhisph.2023.100670.

[pce70049-bib-0084] Urban, J. , R. Bequet , and R. Mainiero . 2011. “Assessing the Applicability of the Earth Impedance Method for In Situ Studies of Tree Root Systems.” Journal of Experimental Botany 62, no. 6: 1857–1869. 10.1093/jxb/erq370. 21273337 PMC3060674

[pce70049-bib-0085] Vanella, D. , S. R. Peddinti , and I. Kisekka . 2022. “Unravelling Soil Water Dynamics in Almond Orchards Characterized by Soil‐Heterogeneity Using Electrical Resistivity Tomography.” Agricultural Water Management 269: 107652. 10.1016/j.agwat.2022.107652.

[pce70049-bib-0086] Wang, H. , E. Zimmermann , M. Weigand , H. Vereecken , and J. A. Huisman . 2023a. “Comparison of Different Inversion Strategies for Electrical Impedance Tomography (EIT) Measurements.” Geophysical Journal International 235, no. 3: 2888–2899. 10.1093/gji/ggad398.

[pce70049-bib-0087] Wang, N. , Y. Li , L. Huang , Z. Y. Wang , and P. F. Zhao . 2023b. “Multi‐Frequency Complex Conductivity Sparse Imaging of Plant Root Zone Based on Space‐Frequency Correlation.” Computers and Electronics in Agriculture 205: 107630. 10.1016/j.compag.2023.107630.

[pce70049-bib-0088] Wasson, A. P. , G. J. Rebetzke , J. A. Kirkegaard , J. Christopher , R. A. Richards , and M. Watt . 2014. “Soil Coring at Multiple Field Environments can Directly Quantify Variation in Deep Root Traits to Select Wheat Genotypes for Breeding.” Journal of Experimental Botany 65, no. 21: 6231–6249. 10.1093/jxb/eru250. 24963000 PMC4223987

[pce70049-bib-0089] Watt, M. , F. Fiorani , B. Usadel , U. Rascher , O. Muller , and U. Schurr . 2020. “Phenotyping: New Windows Into the Plant for Breeders.” Annual Review of Plant Biology 71, no. 1: 689–712. 10.1146/annurev-arplant-042916-041124. 32097567

[pce70049-bib-0090] Weigand, M. , and A. Kemna . 2016. “Debye Decomposition of Time‐Lapse Spectral Induced Polarisation Data.” Computers & Geosciences 86: 34–45. 10.1016/j.cageo.2015.09.021.

[pce70049-bib-0091] Weigand, M. , and A. Kemna . 2017. “Multi‐Frequency Electrical Impedance Tomography as a non‐Invasive Tool to Characterize and Monitor Crop Root Systems.” Biogeosciences 14, no. 4: 921–939. 10.5194/bg-14-921-2017.

[pce70049-bib-0092] Weigand, M. , and A. Kemna . 2019. “Imaging and Functional Characterization of Crop Root Systems Using Spectroscopic Electrical Impedance Measurements.” Plant and Soil 435, no. 1–2: 201–224. 10.1007/s11104-018-3867-3.

[pce70049-bib-0093] Weigand, M. , A. F. Orozco , and A. Kemna . 2017. “Reconstruction Quality of SIP Parameters in Multi‐Frequency Complex Resistivity Imaging.” Near Surface Geophysics 15, no. 2: 187–199. 10.3997/1873-0604.2016050.

[pce70049-bib-0094] Weigand, M. , E. Zimmermann , V. Michels , J. A. Huisman , and A. Kemna . 2022. “Design and Operation of a Long‐Term Monitoring System for Spectral Electrical Impedance Tomography (sEIT).” Geoscientific Instrumentation, Methods and Data Systems 11, no. 2: 413–433. 10.5194/gi-11-413-2022.

[pce70049-bib-0095] Whalley, W. R. , A. Binley , C. W. Watts , et al. 2017. “Methods to Estimate Changes in Soil Water for Phenotyping Root Activity in the Field.” Plant and Soil 415, no. 1–2: 407–422. 10.1007/s11104-016-3161-1. 32025056 PMC6979655

[pce70049-bib-0096] Wilkinson, P. B. , J. E. Chambers , M. Lelliott , G. P. Wealthall , and R. D. Ogilvy . 2008. “Extreme Sensitivity of Crosshole Electrical Resistivity Tomography Measurements to Geometric Errors.” Geophysical Journal International 173, no. 1: 49–62. 10.1111/j.1365-246X.2008.03725.x.

[pce70049-bib-0097] Zamski, E. , and A. Azenkot . 1981. “Sugarbeet Vasculature. I. Cambial Development and the Three‐Dimensional Structure of the Vascular System.” Botanical Gazette 142, no. 3: 334–343. 10.1086/337232.

[pce70049-bib-0098] Zhao, Y. , E. Zimmermann , J. A. Huisman , et al. 2015. “Phase Correction of Electromagnetic Coupling Effects in Cross‐Borehole EIT Measurements.” Measurement Science and Technology 26, no. 1: 015801. 10.1088/0957-0233/26/1/015801.

[pce70049-bib-0099] Zimmermann, E. , and J. A. Huisman . 2024. “The Effect of Heterogeneous Contact Impedances on Complex Resistivity Measurements.” Geophysical Journal International 236, no. 3: 1234–1245. 10.1093/gji/ggad477.

[pce70049-bib-0100] Zimmermann, E. , J. A. Huisman , A. Mester , and S. van Waasen . 2019. “Correction of Phase Errors due to Leakage Currents in Wideband EIT Field Measurements on Soil and Sediments.” Measurement Science and Technology 30, no. 8: 084002. 10.1088/1361-6501/ab1b09.

[pce70049-bib-0101] Zimmermann, E. , A. Kemna , J. Berwix , W. Glaas , and H. Vereecken . 2008. “EIT Measurement System With High Phase Accuracy for the Imaging of Spectral Induced Polarization Properties of Soils and Sediments.” Measurement Science and Technology 19, no. 9: 094010. 10.1088/0957-0233/19/9/094010.

